# Modified hTERT treatment ameliorates pressure overload-induced heart failure

**DOI:** 10.1016/j.ebiom.2026.106203

**Published:** 2026-03-09

**Authors:** Yinlong Zhao, Xiaolu Bao, Weiyao Xiong, Xin Wan, Qingying Yu, Teng Wang, Andrew C.H. Chang, Yangyang Liu, Yanqiu Wang, Ching Shang, Min Wu, Euan A. Ashley, Ming Lei, Junfeng Zhang, Yueheng Wu, Wei Han, Alex C.Y. Chang

**Affiliations:** a Department of Cardiology, Ninth People's Hospital, Shanghai Jiao Tong University School of Medicine, Shanghai, 200125, China; b Shanghai Institute of Precision Medicine, Ninth People's Hospital, Shanghai Jiao Tong University School of Medicine, Shanghai, 200125, China; c Juvensis Therapeutics, Shanghai, China; d Division of Cardiology, Department of Medicine, Stanford University, Palo Alto, CA, USA; e Department of Cardiovascular Surgery, Guangdong Provincial Key Laboratory of South China Structural Heart Disease, Guangdong Cardiovascular Institute, Guangdong Provincial People's Hospital (Guangdong Academy of Medical Sciences), Southern Medical University, Guangzhou, Guangdong, China; f Department of Heart Failure, Heart Center, Shanghai East Hospital, School of Medicine, Tongji University, Shanghai, 200120, China

**Keywords:** Heart failure, hiPSC-CMs, Telomere, DNA damage response, Mitochondria

## Abstract

**Background:**

Heart failure (HF) is a currently incurable disorder that increases the risk for stroke and sudden cardiac death. Shortened telomeres have been linked to the development of cardiomyocyte abnormalities and dysfunction, and telomere reprotection has become a favourable strategy for designing novel heart failure therapies. This study aims to design a pan-HF gene therapy where modified human telomerase expression is driven by cardiac troponin promoter and to evaluate cardiac protection.

**Methods:**

Telomere shortening was determined in cardiomyocytes from *Macaca fascicularis* (cynomolgus monkey) and patients with HF by quantitative fluorescence *in situ* hybridisation (Q-FISH) assays. We bioengineered a catalytic inactivation and nuclear retaining modified human TERT (telomerase reverse transcriptase) gene therapy (AAV9-modhTERT^Y707F, D868A^). In transverse aortic constriction (TAC)-induced WT and myocardial p53 deficient (p53^CKO^) mice HF model, as well as Ang II-induced human induced pluripotent stem cell-derived cardiomyocytes (hiPSC-CMs), we evaluate cardiac protection of modhTERT via echocardiography, RNA-sequence, Western blotting, Proteome Profiler Mouse XL Cytokine Array panel, RT-qPCR, transmission electron microscopy, and immunofluorescence.

**Findings:**

AAV9-modhTERT^Y707F, D868A^ reversed cardiac function decline and prevented onset of cardiac fibrosis in TAC-induced HF murine. At cellular level, modhTERT alleviated contractile dysfunction and aberrant calcium handling in cardiomyocytes isolated from TAC hearts and prevented Ang II-stimulated hiPSC-CMs hypertrophy. Overexpression of modhTERT blocked telomeric DNA damage response (DDR) and p53 ser15-phosphorylation. Myocardial chronic inflammation and reactive oxygen species (ROS) levels were reverted by modhTERT overexpression. Additionally, modhTERT rescued mitochondrial ultrastructure, increased mitochondrial DNA (mtDNA) copy, and restored ATP production through restoration of PGC-1 α and TFAM expression.

**Interpretation:**

We provide evidence that telomere re-protection confers cardiac protection and may serve as a potential gene therapeutic option for treating heart failure.

**Funding:**

This research was supported by the 10.13039/501100001809National Natural Science Foundation of China (82070248, 82300282, 82300476), the Program for Professor of Special Appointment (Eastern Scholar) at Shanghai Institutions of Higher Learning (0900000024), 2023 Shanghai Action Plan for Promoting Scientific and Technological Innovation and Industrial Development of Gene Therapy (23J11900600).

**Research in context**

**Evidence before this study**

Nearly 64 million people worldwide live with heart failure (HF) with a mortality rate of 50% within five years of diagnosis. Current surgical and pharmacological interventions merely slow down progression to extend lifespan but cannot reverse cardiac function. We and others have previously demonstrated that telomere shortening and activation of DNA damage response (DDR) drives heart failure progression. Telomeres are repetitive DNA sequences that are bound by shelterin protein complex at the ends of chromosomes to maintain genomic stability. Telomeres have been perceived to only shorten in proliferating cells due to DNA end replication problem. In stem cells, telomerases bind to telomeric ends and extend telomeres to maintain rejuvenation capacity. We and others have demonstrated that short telomere—through DDR p53 activation which represses mitochondrial biogenesis master regulator PGC1a—can drive cardiac disfunction. Anti-ageing field continues to hunt for better telomerase activating compounds to combat ageing yet overactivation or immortalisation or mutated cells increases the risk of cancer. Thus, using its structural capability to develop a TERT gene therapy to reverse heart failure holds great promise in the fight against heart failure.

**Added value of this study**

In this study, we designed an AAV9 to overexpress catalytically inactive and nuclear-localised telomerase protein (JV101) to re-protect telomeric ends and reverse heart failure. In this pre-clinical efficacy study, we provide both biodistribution and efficacy data of JV101 in TAC heart failure murine models. Single dose of JV101 (4 × 10^12^vg/kg) was sufficient in restoring cardiac function both *in vivo* as well as *in vitro*. Molecularly, JV101 administration rescued telomeric dysfunction, prevented activation of DDR, restored mitochondrial biogenesis and ultrastructure, restored mitochondrial respiration and ATP production, and reversed chronic inflammation.

**Implications of all the available evidence**

JV101 has demonstrated to be efficacious and its unique design may be applied to other forms of heart failure. Provided that cardiomyocytes do not proliferate and there is only limited number of telomeric ends per cardiomyocyte, it is foreseeable that the efficacy of JV101 will be long lasting (lack of dilution due to cell division) and the low dosage will confer great safety profile and an affordable universal heart failure gene therapy.

## Introduction

Heart failure (HF) is a complex clinical syndrome secondary to structural and functional impairments of the heart muscle. Many pathophysiological processes, such as myocyte hypertrophy, desensitisation of β-adrenergic signalling, excitation–contraction coupling changes, and inflammatory response, are involved in heart failure.^1^, ^2^, ^3^ HF has reached epidemic proportions in developed countries, currently affecting over 64.3 million people worldwide.^4^^,^^5^ To improve clinical outcomes and avoid HF, identifying effective therapeutic drugs and molecular intervention targets is needed.

Telomeres, a hallmark of ageing,^6^ are TTAGGG repeat sequences located at chromosomal ends that are bound by shelterin protein complex to maintain genomic stability, and telomere shorten in dividing systems due to DNA replication problem. Shortened telomeres have been linked to genomic instability, activation of DNA damage responses, mitochondrial dysfunction, and reactive oxygen species (ROS) production.^7^^,^^8^ In stem cells, telomerase reverse transcriptase (TERT) proteins are expressed in the nucleus and help maintain replication capacity through telomere elongation during cell division.^9^, ^10^, ^11^ Anti-ageing research has increasingly focused on maintaining telomere length through the activation of TERT to delay ageing, as well as on the prevention and treatment of age-related diseases.^12^, ^13^, ^14^ However, manipulating the telomere system can confer immortalisation thus carries the risk of cancer.^15^^,^^16^ We and others have previously demonstrated that telomere shortening is implicated in cardiovascular disease, despite cardiomyocytes being terminally differentiated post-mitotic cells.^17^, ^18^, ^19^, ^20^, ^21^, ^22^, ^23^ We also demonstrated that short telomeres triggers chromosomal decompaction near telomeric ends, which correlated with an upregulation of cardiomyocyte senescence-related gene transcription.^22^ Thus, TERT gene therapy is a promising candidate that deserves further research efforts for clinical implementation to treat heart diseases.

The notion of using TERT gene therapy to treat cardiovascular disease is supported by the literature. TERT gene therapy improved ventricular function and limited infarct scar size in acute myocardial infarction murine model^24^ and heart failure.^25^, ^26^, ^27^ Moreover, TERT has also been demonstrated to localise and play a different biological role in mitochondria.^28^, ^29^, ^30^, ^31^ Mitochondrial TERT reduces infarct size and improves mitochondrial respiration after myocardial infarction, attenuates pathological cardiac hypertrophy,^32^^,^^33^ and rescues doxorubicin-induced cardiotoxicity.^25^ Previous studies indicated that hydrogen peroxide stress can induce Src-dependent tyrosine 707 phosphorylation of TERT and drive nuclear export of TERT.^34^^,^^35^ Mutations in the conserved residues K902, R631, K626, D712, D868, and D869 are known to inhibit the catalytic activity of the human telomerase protein.^36^, ^37^, ^38^, ^39^ D868 at the catalytic center is the most important catalytic activity residue.^40^^,^^41^ Here, we engineered a catalytically inactive and nuclear retaining human telomerase protein (modhTERT^Y707F, D868A^) and tested whether telomere reprotection is capable of ameliorating heart failure progression.

In this study, we generated a cardiac troponin t promoter driven AAV9-modhTERT^Y707F, D868A^ tested its efficacy in heart failure models. We probed the biodistribution of modhTERT^Y707F, D868A^ in human induced pluripotent stem cell derived cardiomyocytes (hiPSC-CMs) and in transverse aortic constriction (TAC) murine hearts when administered through intravenous injection. We evaluated whether modhTERT^Y707F, D868A^ conferred efficacy through reprotection of telomeric ends. We examined downstream molecular pathways including DNA damage response (DDR), p53 activation, and mitochondrial biogenesis (PGC1 α and TFAM). Histology, cardiac inflammation, and level of reactive oxygen species (ROS) were also examined.

## Methods

### Ethics

Human heart tissue samples were obtained from patients with end-stage HF that received heart transplantations or from unused donor hearts in an Institutional Review Board-approved protocol at Guangdong General Hospital (Institutional Review Board No. GDREC2016255H). The collection and research use of these tissues was approved by the Ethics Committee of Guangdong General Hospital. Informed consent was obtained from all the participants. The HF of non-human primates (NHPs) was induced by doxorubicin (ADR) and acute myocardial infarction (AMI). Non-human primate hearts were acquired from Shanghai Prisys Biotechnologies Co., Ltd (Shanghai, China), and all animal protocols in this study were approved by the Animal Experiment Ethics Committee of Shanghai Ninth People's Hospital (SH9H-2021-TK500-1). Human iPSC lines were generated and catalogued as part of Stanford Cardiovascular Institute Biobank governed by scientific and ethics committee. Written consent from patients were collected for to use their biological samples for research and subsequent hiPSC generation. hiPSC lines were obtained from Stanford through a MTA agreement and all protocols using human iPSC were reviewed and approved by the Ethics Review committee at Ninth People's Hospital, Shanghai Jiao Tong University School of Medicine (SH9H-2018-T83-3).

### Design of AAV9-modhTERT^Y707F, D868A^

To minimise tumorgenicity and restrict telomerase protein to localise in the nucleus of cardiomyocytes, we introduced catalytic inactivation (D868A) and nuclear export phosphorylation (Y707F) mutations in of GOI design. Adeno-associated virus 9 (AAV9) viruses containing either a haemagglutinin (HA)-tagged hTERT^Y707F, D868A^ (AAV9-HA-modhTERT^Y707F, D868A^) under the control of the cardiomyocyte-specific cardiac troponin T (cTnT) promoter were designed and produced using HEK 293T cells using a three-plasmid system. GMP grade virus production was contracted to PackGene Biotech, China. Sequence conservation of TERT between different model organisms was evaluated by BLAST analysis.

### Human iPSC culture and cardiac differentiation

We are grateful to David L. Mack and Martin K. Childers for providing the hiPSC lines.^42^, ^43^, ^44^ Healthy human hiPSC (UC3.4) lines (obtained from University of Washington, Supplementary Table S1) were used for cardiomyocyte differentiation. Human-induced pluripotent stem cells (hiPSCs) were cultured in Nutristem hPSC XF medium (Biological Industries) in 6-well plates coated with Matrigel (Corning, 356231). 5 μM Rock inhibitor (Y-27632 dihydrochloride; Selleckchem, S1049) was added to the cell culture on the first day and removed by replacing fresh medium after one day. For cardiac differentiation, hiPSCs differentiation was induced to generate beating CMs at 70–90% confluency as described previously.^42^ Briefly, hiPSC were treated with 4–6 μM CHIR-99021 (Selleck Chemicals, S2924) for 2 days, followed by a Wnt inhibitor IWR-1 (5 μM; Sigma, I0161) treatment for another 2 days, in RPMI 1640 medium supplemented with B27 minus insulin (Thermo Fisher Scientific, A1895601). On day 5, medium was changed to fresh RPMI 1640 medium supplemented with B27 minus insulin for 2 days and replaced to RPMI 1640 medium supplemented with B27 until day 10. hiPSC-CMs were then purified using a metabolic selection medium which consisted of RPMI 1640 without glucose, B27 supplement (Thermo Fisher Scientific, 17504044) and 4 mM of sodium dl-lactate (Sigma, 72-17-3). The cardiomyocytes were maintained for an extended time frame with medium changes every two days. The mycoplasma test was negative. After treatment of 10 μM Ang II for 24 h, hiPSC-CMs were treated with 10 MOI either JV101 or Vector viral particles for 48 h hiPSC-CMs were harvested after 72 h for subsequent experiments.

### Transverse aortic constriction (TAC) model of cardiac remodelling in mice

8 weeks old male C57BL/6J mice obtained from GemPharmatech Co., Ltd. p53^CKO^ (Myh6-Cre x p53flox/flox) were generated inhouse as previously described.^45^^,^^46^ To minimise hormonal fluctuations, we only used male mice in the animal experiments. All animals were housed with 12- hour light/dark cycle, a constant room temperature, and fed a standard rodent diet (Jiangsu Xietong Pharmaceutical Co., Ltd, 1010001). Mice were acclimatized to the laboratory for 2 weeks before initiating the studies. Detailed methods for these models are described below.

Mice were randomly divided into three groups: untreated mice (Sham, C57BL/6J mice n = 6, p53^CKO^ mice n = 5); TAC mice receiving Vector (TAC + Vector, C57BL/6J mice n = 8–10, p53^CKO^ mice n = 10); TAC mice treated with JV101 (TAC + JV101, C57BL/6J mice n = 8–10, p53^CKO^ mice n = 7). To perform TAC, mice were anaesthetised by intraperitoneal injection of 1.2% 2,2,2-tribromoethanol solution (0.2 mL/10 g body weight). Mice were placed in a supine position and an endotracheal tube was inserted. Then the mice were ventilated using a volume-cycled rodent ventilator with a tidal volume of 0.4 mL room air and a respiratory rate of 110 breaths/minute. The chest cavity was exposed by cutting open the proximal portion of the sternum. After the aortic arch between the innominate and left common carotid arteries was isolated, it was constricted with a 7-0 nylon suture tied firmly 3 times against a 25-gauge blunted needle for TAC. The needle was immediately withdrawn after the ligation. Sham-operated mice were subjected to identical interventions except for the constrictions of the aorta. Vector and JV101 treatments were initiated 15 days after surgery. After echocardiographic analysis at 8 weeks, mice were sacrificed by cervical dislocation, and hearts were removed and weighed promptly. Survival studies were also conducted on wild-type Sham, TAC, and TAC mice treated with JV101 (4 × 10^12^ vg/kg) or Entresto (82 mg/kg/day), or JV101+Entresto (n = 23 animals per group).

### Echocardiographic assessment of mouse heart function

For cardiac function, echocardiography was performed before and 4 weeks after 8 weeks after TAC surgery at a weekly frequency using a Visual Sonics Vevo 3100 system (Visual Sonics, FUJIFILM). In brief, mice were anaesthetised use 2% isoflurane and maintained the heart rate use 0.5%–1.0% isoflurane. Systolic functions were measured at the midventricular long-axis using M-mode scanning while maintaining the heart rate at the range of 425–475 beats per minute. Technical triplicates were averaged per animal and the means were used for subsequent statistical analyses.

### AMCMs isolation

Adult mice were heparinized and anaesthetised using deep isoflurane (5%) anaesthesia. The hearts were surgically isolated, washed in ice-cold cardiomyocyte isolation buffer (CIB; 120 mM NaCl, 5.4 mM KCl, 0.5 mM MgSO_4_, 0.33 mM NaH_2_PO_4_, 25 mM NaHCO_3_, 22 mM glucose, 25 mM HEPES, 10 mM BDM, and 30 mM taurine), cannulated via the ascending aorta and digested through perfusion with an enzyme CIB buffer containing 1 mg/mL Type II Collagenase (BioFroxx, 2275) and 0.6 mg/mL Type IV Collagenase (BioFroxx, 2091) for 15 min at 37 °C using a Langendorff perfusion system. The digested hearts were mechanically shredded and dissociated in Minimum Essential Medium Eagle (Sigma–Aldrich) supplemented with 10% bovine serum albumin (BSA; Sigma–Aldrich, V900933). After filtration with a 100 μm mesh filter and centrifugation at 500 rpm for 5 min, harvested adult mouse cardiomyocytes (AMCMs) were reintroduced with calcium in four concentration gradients, ranging from 0 to 900 μm. Newborn C57BL/6J mice (within 72 h) were euthanised by decapitation and the hearts were collected.

### Histological analysis

Tissues were removed and fixed in 4% formalin (pH 7.4) overnight. After a series of ethanol dehydration, samples were embedded in paraffin. Heart sections that were embedded in paraffin were stained using haematoxylin and eosin (H&E) and Masson's trichrome to observe the cardiomyocyte hypertrophy and fibrosis. Wheat germ agglutinin (WGA, Solarbio, I3300) staining was used to determine the cross-sectional area (CSA) and phalloidin (Proteintech, PF00003) staining was used to determine the cell morphological changes of cardiomyocytes. The histological features were observed and captured under a light microscope (Olympus). We also performed the quantitative assessment of the fibrosis level and cardiomyocytes cross-sectional area with Image Pro Plus (v6.0).

### Immunofluorescence staining

The cells were fixed with 4% paraformaldehyde and permeabilized with 0.1% Triton X-100 in PBS. After blocking with 1% BSA (0.3% Triton X-100/0.1% Tween-20), then they were incubated with different primary antibodies. AAV9-HA-hTERT^Y707F, D868A^ expression and localisation in hiPSC-CM and mice were investigated using the rabbit anti-rabbit-HA-Tag antibody (1:200, Cell Signalling Technology, 3724S), followed by incubation with Alexa Fluor 594 (1:1000, Thermo Fisher Scientific, A11032) as corresponding secondary antibody. Besides, anti-rabbit-Ki67 (1:200, Abcam, ab15580), were used as primary antibodies, followed by incubation with corresponding secondary antibody including Alexa Fluor 488 (1:1000, Thermo Fisher Scientific, A11008), Nuclei were counterstained with DAPI (1 mg mL^−1^) (Sigma–Aldrich, D9542). For image acquisition, a Zeiss LSM880 confocal microscope was used.

### Live cell imaging

In AMCMs, mitochondria were stained by Mito-tracker Green (Invitrogen, M7514) and intracellular ROS staining (Thermo Fisher Scientific, C10443). Hoechst (Solarbio, C0031) nuclei staining was used for normalisation. All living cell dyes were used according to the manufacturer's instructions. Fluorescence intensities were captured and quantified using an Operetta CLS High Content Imaging System (PerkinElmer). All results were presented as relative intensity using vehicle group for normalisation.

### Telomere Q-FISH and immuno-FISH

For hiPSC-CMs, cells were replated onto Matrigel-coated eight-chamber slides and fixed with 4% paraformaldehyde. Paraffin-embedded heart sections from humans, *Macaca fascicularis*, and mice were deparaffinized in xylene (Sigma–Aldrich, Missouri, USA) and dehydrated in an ethanol series. Telomere Q-FISH was performed as previously described by using TelC-Cy3 PNA probe (CCCTAACCCTAACCCTAA; PNA Bio).^19^ For immune-FISH, tissues or cells were further blocked with staining buffer (20% FBS/0.1%Triton X-100/PBS solution) and stained with prediluted primary antibody: anti-rabbit-53BP1 (1:200, Cell Signalling Technology, 4937S), anti-mouse-cardiac troponin T (1:400, Abcam, ab8295) and anti-rabbit-HA-Tag antibody (1:200, Cell Signalling Technology, 3724S) for 4 h at room temperature in staining buffer. After washed with PBS solution, samples were incubated with secondary antibodies for 1 h and counterstained with DAPI (4 μg/mL) in PBS solution for 15 min, washed, air-dried, and mounted with VECTASHIELD ® Mounting Medium (Vectorlabs). For cells, confocal images were acquired as stacks for a total of 4 μm, while tissue confocal images were acquired as stacks every 1 μm for a total of 5 μm using a Zeiss LSM880 confocal microscope using a 60× objective, and maximum projections were performed using the Zen software. Telomere length was determined by PNA signal intensity and quantified using Imaris (Bitplane).

### Senescence-associated β-galactosidase (SA-β-gal) activity

SA-β-Gal activity was assessed using a senescence associated β-galactosidase staining kit (Solarbio). In brief, cells were fixed with fixation buffer containing formaldehyde for 10 min. Next, the cells were treated with staining buffer containing X-gal overnight at 37 °C. Stained cells were washed using PBS and observed by Zeiss LSM880 confocal microscope.

### Nuclear and cytoplasmic protein extraction

Transfected cells were washed thrice with phosphate-buffered saline, then suspended in 600 μl of cytoplasmic extraction reagent I (CER I) buffer (NE-PER kit, Thermo Scientific). Nuclear proteins were extracted in accordance with the manufacturer's instructions. Protein concentrations were determined using the Bradford method (Bio-Rad SmartSpec Plus, Bio-Rad Laboratories, Inc., USA).

### Immunoblotting

Proteins were extracted from hiPSC-CMs and AMCMs with radioimmunoprecipitation buffer containing protease and phosphatase inhibitors. Lysates were centrifugated at 12,500 rpm for 15 min at 4 °C and the supernatant was collected and transferred to a new Eppendorf tube. The protein concentrations were determined using a BCA kit (Thermo Fisher Scientific, 23227). The lysate was subjected to SDS-PAGE and transferred to polyvinylidene fluoride (PVDF) membrane. Membranes were blocked with PBS supplemented with 5% (w/v) non-fat dry milk (NFDM) for 1 h, and incubated with primary antibodies overnight in antibody dilution buffer (TBS supplemented with 0.01% (v/v) Tween-20 and 5% (w/v) BSA). The primary antibodies: histone H_2_AX (1:1000, Cell Signalling Technology, 7631S), γH_2_AX (1:1000, Cell Signalling Technology, 9718S), 53BP1(1:1000, Cell Signalling Technology, 4937S), p53 (1:1000, Cell Signalling Technology, 2524S), Phospho-p53 (Ser15) (1:1000, Cell Signalling Technology, 9284s), PGC-1α (1:1000, Santa Cruz, sc-517380), TFAM (1:1000, Proteintech, 22586-1-AP), TRF1 (1:1000, Abcam, ab192629), RAP1 (1:1000, Santa Cruz, sc-53434), TIN2 (1:1000, Proteintech, 11368-1-AP), POT1 (1:1000, Novus Biologicals, NB500-176), HA-tag (1:1000, Cell Signalling Technology, 3724s), DYKDDDDK Tag (1:1000, Cell Signalling Technology, 14793s), H3 (1:1000, Cell Signalling Technology, D5A7), GAPDH (1:1000, Proteintech, 60004-1-Ig) followed by secondary antibody for 1 h with a 1:2000 dilution of IgG Goat Anti-Mouse HRP (Proteintech, SA00001-1) or IgG Goat Anti- Rabbit HRP (Proteintech, SA00001-2). Western blots were developed using ECL chemiluminescent substrate (Thermo Fisher Scientific, 34098CN). The results were visualised using an Amersham Imager 600 (General Electric Company).

### RNA isolation and real time-quantitative PCR (RT-qPCR)

Total RNA was extracted from isolated cardiomyocytes using TRIzol (Invitrogen, 15596-026) according to the manufacturer's instructions. 100 ng RNA was used to generate cDNA by using the HiScript® II 1st Strand cDNA Synthesis Kit (Vazyme, R201-01/02). RT-qPCR was performed on LightCycler @ 480 II (Roche) using a ChamQ Universal SYBR qPCR Master Mix (Vazyme). The relative mRNA expression levels were calculated by the 2^−ΔΔCt^ method and normalised to GAPDH. Sequences of primers used are listed in Supplementary Table S2.

Real-time quantitative polymerase chain reactions were carried out on an ABI QuantStudioTM 6 Flex machine (Applied Biosystem). Inventoried TaqMan FAM probes (Supplementary Table S2) were used for the relative quantification of the TERT DNA copy number in different tissues with TaqMan™ Gene Expression Master Mix Kit (Applied Biosystems™, 4369016) according to the manufacturer's instructions.

### Mitochondria DNA copy number analysis

For mtDNA copy number assay, 100 ng total DNA was isolated using the cardiac tissue or cells Genome DNA Extract kit (Solarbio, D1700) according to the manufacturer's instructions. Primers for MTRNR2, MTTL1, β-Globin human gene and mt- Cytb, β-actin mouse gene (Supplementary Table S2) were used. Real-time quantitative polymerase chain reactions were carried out on an ABI QuantStudioTM 6 Flex machine (Applied Biosystem).

### Transmission electron microscopy

Heart tissue and hiPSC-derived cardiomyocytes were fixed using 2.5% glutaraldehyde solution for 2 h at room temperature in dark, washed three times with 0.1 M PBS, and post-fixed with 1% osmium tetroxide for 2 h at 4 °C. Next, samples were washed with 0.1 M PBS for three times, then dehydrated in graded ethanol (30%, 50%, 70%, 85%, 95%, 100%) each for 30 min, and infiltrated with ethanol: Epon 812 (2:1, 1:1, 1:2, each for 30 min). Next, samples were embedded with Epon resin, sectioned at 70 nm thickness. The thin sections were mounted onto formvar-coated copper grids, counterstained with 3% uranyl acetate in 70% methanol and 30% water for 7 min, followed by lead citrate for 3 min. Micrographs were captured using a FEI (Tecnai G2 Spirit 120 kV) electron microscope (FEI Italia, Italy). For morphometric analysis of mitochondrial: mitochondrial area and cristae number were measured using the Image J Multi-measure plug-in.

### Total ATP measurement

Total ATP levels of mouse heart tissue and hiPSC-CMs were measured with ATP Assay Kit (Beyotime, S0026) according to the manufacturer's instructions. Each heart sample was collected from the left ventricle apex.

### NT-proBNP and profiler mouse XL cytokine array panel

The serum levels of NT-proBNP were measured using Mouse NT-proBNP ELISA Kit (Thermo Fisher Scientific, EEL193), according to the manufacturer's instructions. Briefly, this essay employs a quantitative sandwich enzyme technique. A microplate is pre-coated with an antibody specific for NT-proBNP; the analyte is captured and then sandwiched with a biotin-conjugated antibody. Thereafter, avidin-conjugated horseradish peroxidase is added, followed by a particular substrate. Finally, colour development is stopped, and the intensity of the colour is measured. In our assay system, the intensity of the developed colour was positively correlated with the concentration of mouse NT-proBNP in the sample, and the absorbance (OD value) was measured using an absorbance microplate reader (SpectraMax plus 384, American Molecular Devices Scientific Company) at 450 nm wavelength.

To examine the effects of JV101 on chronic inflammation, an inflammatory cytokine array (RD. ARY028, R&D Systems) was used according to manufacturer's instructions to analyse actual concentration of inflammatory proteins in cardiac tissue from mouse models.

### RNA-sequencing

3−4 × 10^5^ AMVMs from single hearts were Langendorff isolated and lysed in Trizol for RNA extraction. RNA-seq using AMVMs isolated from Sham, TAC, and TAC + JV101 were performed. After RNA quantification and quality check, a total amount of 1 μg RNA per sample was used to generate sequencing libraries using NEBNext® UltraTM RNA Library Prep Kit for Illumina® (NEB). Briefly, 150 bp paired-end reads were processed and generated, the libraries were sequenced on an Illumina HiSeq platform. Differential gene expression analysis was done using the R package DEseq2 (v 1.6.3). P value < 0.05 and fold change >2 or <0.5 was set as the threshold for significantly differential expression. Hierarchical cluster analysis of DEGs was performed to demonstrate the expression pattern of genes in different groups and samples. GO enrichment and KEGG pathway enrichment analysis of DEGs were performed respectively using R based on the hypergeometric distribution.

### Statistical analysis

All quantitative data are shown as Mean ± SEM. Statistical differences among groups were analysed by One-way ANOVA with Tukey's multiple comparisons test. Statistical significance between two groups was determined using two-tailed Student's t -test. All statistical analyses were performed using GraphPad Prism 9.0 software (GraphPad Software, version 9.0). Statistical significance was considered. Kaplan–Meier survival analysis was performed to generate survival curves, and the log rank test was used to calculate *P*-values, ∗*P* < 0.05 was considered statistically significant.

### Role of funders

The funders provided financial support for this study and were not involved in the study design, data collection, data interpretation, or writing of the manuscript.

## Results

### Myocardial shortened telomeres negatively correlate with left ventricular ejection fraction (LVEF %) in non-human primate and human hearts

**Fig. 1:**
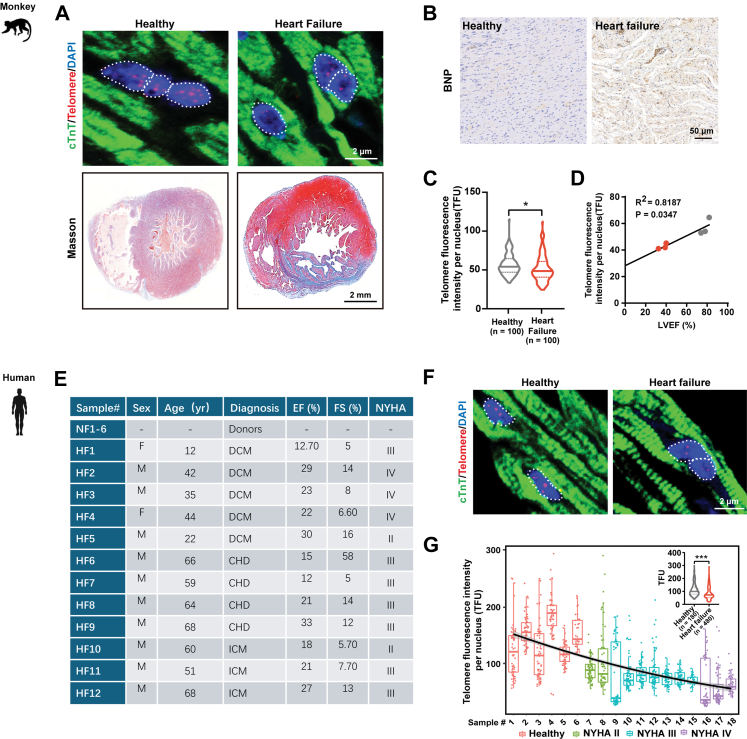
**Human and non-human primate (NHP) failing hearts exhibit myocardial telomere shortening and LVEF decline.** (A) Paraffin-embedded NHP cardiac samples were used for telomere Q-FISH quantification of CMs. NHP CMs (cardiac Troponin-T [green]) were stained for telomere (red) and for nuclear DAPI (blue) in heart failure and control cardiac tissue sections. Masson's trichrome staining was performed to confirm cardiac fibrosis in failing NHP hearts (healthy NHP, n = 3; HF NHP, n = 3). (B) Immunohistochemistry for heart failure marker BNP (brown) in HF and control cardiac tissue sections was performed. (C) Average NHP myocardial telomere fluorescence intensities relative to the nucleus signal (TFU) are shown. (D) Pearson correlation coefficients between mean TFU and left ventricular ejection fraction (LVEF) in NHP hearts. (E) Clinical diagnosis of healthy (n = 6 biopsies from nonfailing donor hearts, NF) and heart failure (n = 12 for heart failure, HF) patient samples classified by New York Heart Association (NYHA) are shown. DCM, dilated cardiomyopathy; CHD, coronary heart disease; ICM, ischaemic cardiomyopathy. (F) Paraffin-embedded cardiac samples were used for telomere Q-FISH quantification of CMs. Patient CMs (cardiac Troponin-T [green]) were stained for telomere (red) and for nuclear DAPI (blue) in patient and control cardiac tissue sections. (G) Telomere fluorescence intensities relative to the nucleus signal (TFU) per patient and best fit curve are shown (n = 18). Data are represented as mean ± SEM. ∗*P* < 0.05, ∗∗∗*P* < 0.001. Statistical analysis for the comparison of two groups was performed using two-tailed unpaired Student's t-test.

To understand the role of the telomere in the heart, we first examined myocardial telomere lengths in non-human primate (NHP; *M. fascicularis*) with heart failure (HF). In HF samples, NHP hearts exhibited an increase of cardiac fibrosis and elevated levels of brain natriuretic peptide (BNP) by Masson trichrome staining and immunohistochemistry, respectively (Fig. 1A and B). Quantitative fluorescent *in situ* hybridisation (Q-FISH) using TelC probe against telomeric repeats showed a significant decrease in telomere fluorescence unit per nucleus (TFU) in HF compared to control NHP cardiomyocytes (Fig. 1A and C). And short telomeres correlated with a more reduced left ventricular ejection fraction (LVEF %) (Fig. 1C and D). Furthermore, a total of 6 healthy and 12 heart failure patient tissue samples ranging from New York Heart Association (NYHA) class II to IV were evaluated (Fig. 1E). In keep, the telomere length in human failing hearts correlated negatively with NYHA II to IV classification (Fig. 1F and G). These results demonstrated the presence of myocardial telomere attrition in NHP and human failing hearts.

### Design of AAV9-modhTERT^Y707F, D868A^ to target myocardial telomeres

To design a gene therapy that overexpresses a modified telomerase protein that targets telomeric ends, catalytic inactivation (D868A, minimise tumorgenicity) and blocked nuclear export (Y707F, localise in the nucleus) mutations were introduced. Given the lack of a good commercial TERT antibody, we first generated an adeno-associated virus 9 (AAV9) vector to overexpress the haemagglutinin (HA)-tagged hTERT^Y707F, D868A^ (AAV9-HA-modhTERT^Y707F, D868A^; JV101) under control of the cardiomyocyte-specific cardiac troponin T (cTNT) promoter (Supplementary Figure S1A). Wild type (WT) mice receiving intravenous injection of 4 × 10^12^ vector genomes per kilogramme (vg kg^−1^) showed no signs of toxicity and were vital. At 3 weeks post-injection, the Langendorff-isolated heart showed stable nuclear HA-modhTERT^Y707F, D868A^ proteins in cardiomyocytes but not fibroblasts (Supplementary Figure S1B and S1C). Echocardiography was performed to monitor cardiac functional changes when animals were injected with HA-JV101. Compared with wild type (WT) mice and vector mice, the ejection fraction (EF) and fractional shortening (FS) were not altered in HA-JV101 treated mice (Supplementary Figure S1D and S1E).

**Fig. 2:**
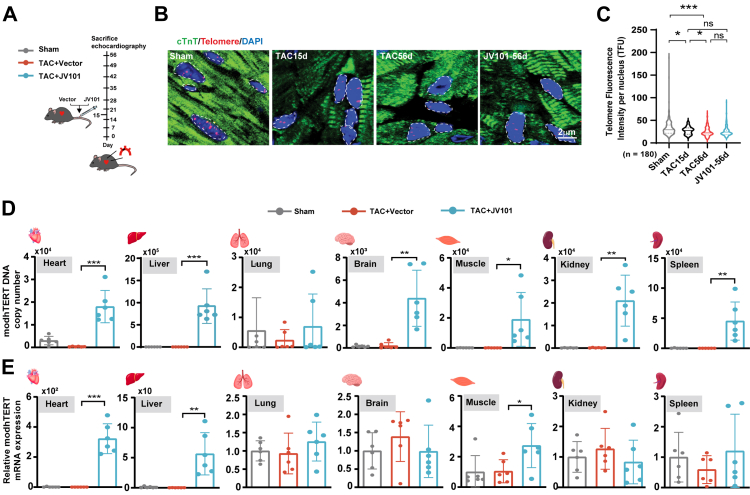
**AAV9-modhTERTY707F, D868A exhibits a good biodistribution profile but does not reverse telomere attrition.** (A) Schematic diagram of experimental design. Once TAC mice enter heart failure stage (decrease in LVEF by 20%, day 14), mice were randomly divided and intravenously injected with either JV101 or vector (4 × 10^12^vg/kg vector genome copies [vg/kg]). (B and C) Paraffin-embedded Ang II mice and control cardiac samples were used for telomere Q-FISH quantification of CMs. Cardiomyocytes (cardiac Troponin-T [green]) were stained for telomere (red) and for nuclear DAPI (blue). (D) Viral DNA copy numbers in various organs were assayed using TaqMan RT-qPCR. (E) Relative expression levels of modhTERT^Y707F, D868A^ mRNA in various organs were assayed using RT-qPCR. Data are mean ± SEM. ∗*P* < 0.05, ∗∗*P* < 0.01, ∗∗∗*P* < 0.001. Statistical differences among groups were analysed by one-way ANOVA followed by Tukey's multiple comparisons test.

To evaluate the therapeutic potential of JV101 in heart failure, TAC surgeries were performed on 8-week-old wild-type mice, cardiac dysfunctional animals were injected with JV101 on day 15, and cardiac function was continuing to be monitored by echocardiography until day 56 (Fig. 2A). A dose titration pilot experiment was carried out to determine the optimal dosage (low: 2 × 10^12^vg/kg; medium: 4 × 10^12^vg/kg; high: 2 × 10^13^ vg/kg) and a larger cohort receiving either JV101 (medium dose: 4 × 10^12^vg/kg) or vector control through tail vein intravenous injection on day 15 was performed to evaluate efficacy (Supplementary Figure S2A and S2B). To examine degree of telomere shortening at the time of JV101 administration, Q-FISH was performed on heart sections from Sham, day 15 TAC hearts, day 56 TAC and TAC + JV101 hearts. Compared to Sham hearts, day 15 TAC cardiomyocytes already exhibited telomere shortening which was further worsened on day 56 (Fig. 2B and C). In comparison, JV101 treatment (day 15 TAC versus day 56 TAC + JV101) arrested myocardial telomeres from further shortening (Fig. 2B and C). Biodistribution assessment was carried out for both viral DNA as well as mRNA in murine heart, liver, lung, brain, muscle, kidney, and spleen. JV101 viral particles (DNA) were detected in all examined tissues except for the lungs (Fig. 2D and Supplementary Excel1). By mRNA RT-qPCR analysis, cTNT promoter demonstrated strong cardiac specificity but with expression leakage in the liver and muscle (Fig. 2E), is in keep with previous reports.^47^^,^^48^ Together, we confirmed that AAV9-modhTERT^Y707F, D868A^ exhibits a favourable biodistribution profile but is insufficient to reverse telomere shortening.

### JV101 treatment protects against pressure overload–induced heart failure *in vivo*

**Fig. 3:**
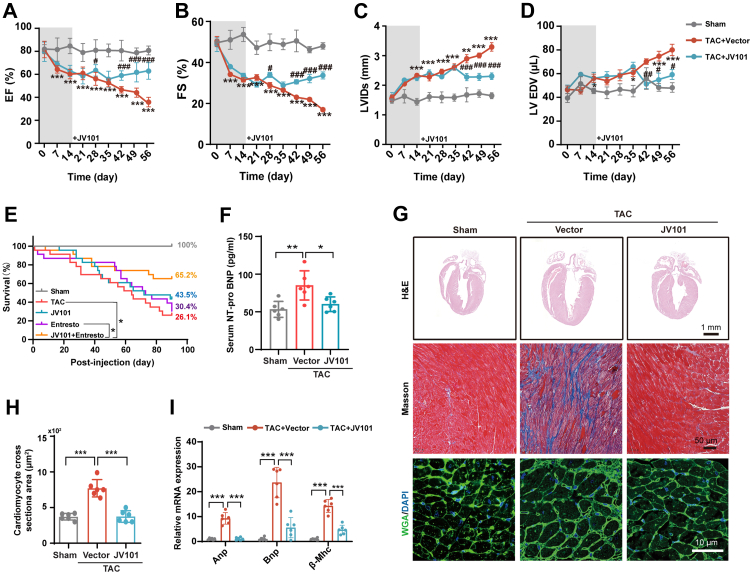
**Single dose of JV101 prevented cardiac dysfunction and blocked cardiac fibrosis in TAC-induced heart failure mice.** (A–D) Cardiac function evaluated by echocardiography. Left ventricular ejection fraction (EF %), fractional shortening (FS %), left ventricular internal diameter at end-systole (LVIDs), and left ventricular end-diastolic volume (LV EDV) at 0, 7, 14, 21, 28, 35, 42, 49, and 56 days are shown. (Sham, n = 6; TAC + Vector, n = 8–10; TAC + JV101, n = 8–10; significance between TAC + Vector vs. saline [∗] and TAC + JV101 vs. TAC + Vector [#] are shown). (E) Precent survival of Sham, TAC, JV101, Entresto, and JV101+ Entresto mice. (F) Serum NT-pro BNP concentration of Sham, TAC and TAC + JV101 mice. (G) Haematoxylin and eosin (longitudinal), Masson trichrome (longitudinal), and wheat germ agglutinin (transverse) micrographs are shown. (H) Quantitative analysis of cardiomyocyte cell sizes from transverse WGA staining (n = 6 animals each). (I) Expression levels of *Anp*, *Bnp*, and *β-Mhc* in primary cardiomyocytes were determined by RT-qPCR (cardiomyocytes from n = 6 animals each). Data are represented as mean ± SEM. ∗*P* < 0.05, ∗∗*P* < 0.01, ∗∗∗*P* < 0.001; ^#^*P* < 0.05, ^##^*P* < 0.01, ^###^*P* < 0.001. Statistical differences among groups were analysed by one-way ANOVA followed by Tukey's multiple comparisons test.

In TAC animals, cardiac hypertrophy developed after 7 days, as shown by an increase in left ventricular (LV) posterior wall thickness at end-diastole (Supplementary Table S3). A reduction in LV ejection fraction and LV fractional shortening, as well as an increase in LV internal diameter at end-systole (LVIDs) was seen on day 7 post-TAC surgery and animals progress into HF on day 14 (Fig. 3A–C). Compared to the vector group, JV101 treated TAC animals exhibited an improvement in LV ejection fraction (EF) and fractional shortening (FS) by day 42 (Fig. 3A and B). Structurally, left ventricular internal diameter at end-systole (LVIDs) and left ventricular end-diastolic volume (LV EDV) were reversed in JV101-treated TAC mice compared to vector-TAC animals (Fig. 3C and D). By day 56, JV101 treatment rescued cardiac structure including heart diameter, volume, stroke volume, cardiac output, left ventricular posterior wall thickness, but not left ventricular anterior wall thickness (Supplementary Table S3). Blood routine and blood biochemistry analyses showed no immunogenicity activation at 56 days (Supplementary Tables S4 and S5). Entresto, an angiotensin receptor-neprilysin inhibitor (ARNI), is the standard of care providing mortality and morbidity benefits in adult patients with chronic HF and reduced left ventricular ejection fraction.^49^ In a head-to-head comparison, a single dose of JV101 treatment raised the percentage survival of TAC animals from 26.1% to 43.5% while Entresto treated were 30.4%. When used in combination, 65.2% TAC animals survived (Fig. 3E). The heart weight to body weight (HW/BW), HW to tibia length ratios, and NT-proBNP levels were all significantly increased after TAC surgery, but JV101 treatment prevented this change (Supplementary Figure S2C and S2D, Fig. 3F). Furthermore, JV101 treatment significantly prevented cardiac fibrosis detected by Masson staining (Fig. 3G), blocked myocardial hypertrophy as measured by myocardial cross section area (Fig. 3G and H), and reduced expression of hypertrophic genes (*Anp, Bnp, β-Mhc*; Fig. 3I). Together, these data indicated that a single intravenous injection of JV101 can rescue cardiac dysfunction in TAC-induced heart failure.

### JV101 rescues contractile dysfunction in TAC murine cardiomyocytes and Ang II- stimulated hiPSC-CMs hypertrophy

**Fig. 4:**
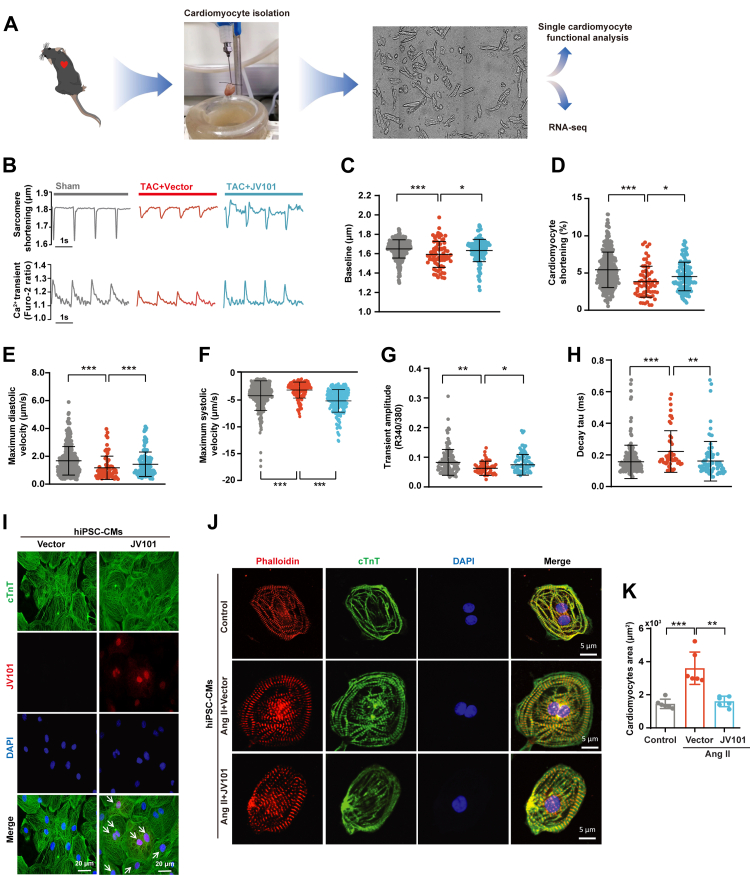
**JV101 treatment alleviates isolated AMCMs dysfunction from TAC mice and Ang II-induced hiPSC-CMs hypertrophy.** (A) Schematic diagram of Langendorff-perfused isolated adult mouse cardiomyocytes (AMCMs). Evaluated a single cardiomyocyte and performed RNA-seq. (B) Representative recordings of sarcomere shortening and Ca^2+^ handling traces are shown (mice n = 5 per group; cardiomyocytes n = 100–120 per group). (C–F) Quantification of sarcomere baseline, cardiomyocyte shortening, maximum diastolic velocity, and maximum systolic velocity of isolated cardiomyocytes are shown. (G and H) Quantification of calcium transient amplitudes and decay tau are shown. (I) Representative micrographs of hiPSC-CMs displaying nuclear HA-modhTERT^Y707F, D868A^ (red) after AAV9-modhTERT^Y707F, D868A^ infection (cTNT [green] and DAPI [blue]). (J) Representative micrographs of hiPSC-CMs displaying Phalloidin (red), cTNT (green) and DAPI (blue). (K) Quantification of cell size from Phalloidin staining. Data are represented as mean ± SEM. ∗*P* < 0.05, ∗∗*P* < 0.01, ∗∗∗*P* < 0.001. Statistical differences among groups were analysed by one-way ANOVA followed by Tukey's multiple comparisons test.

Next, adult mouse cardiomyocytes (AMCMs) from TAC and JV101-treated animals were isolated using Langendorff perfusion and assayed using an Ionoptix HTS system (Fig. 4A). AMCMs in each group were stimulated at 1.0 Hz to elicit cell sarcomere shortening and calcium transients (Fig. 4B). TAC AMCMs exhibited marked impaired sarcomere shortening, as revealed by a decrease in baseline (Fig. 4C), sarcomere length shortening amplitude (Fig. 4D), maximum diastolic velocity (Fig. 4E), and maximum systolic velocity (Fig. 4F), which were ameliorated by JV101 treatment. Ca^2+^ flux measurement showed enhanced Ca^2+^ transient amplitude in JV101-treated AMCMs compared to TAC AMCMs (Fig. 4G). The time constant of the Ca^2+^ transient decay was accelerated in JV101-treated AMCMs, indicating enhanced AMCMs function (Fig. 4H).

Human induced pluripotent stem cells (hiPSC) were differentiated into beating cardiomyocytes (hiPSC-CMs) as previously demonstrated^42^ (Supplementary Table S1). First, we infected hiPSC-CMs with JV101 and confirmed the nuclear localisation of HA-modhTERT proteins by cTNT and telomere Q-FISH immunofluorescence co-staining (Fig. 4I). Ang II treated hiPSC-CMs displayed an increase in the hiPSC-CMs area, indicative of pathogenic hypertrophy, but this was absent in the JV101-treated hiPSC-CMs (Fig. 4J and K).

### JV101 alleviates heart failure by preventing telomeric DNA damage, mitochondrial dysfunction and chronic inflammation

**Fig. 5:**
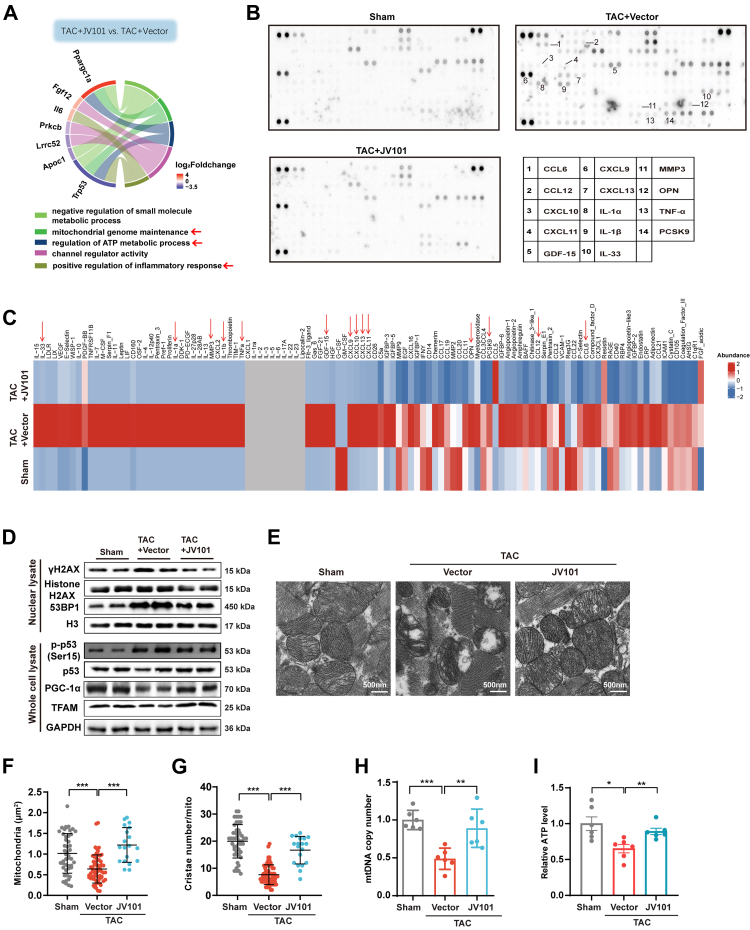
**JV101 inhibited telomeric DNA damage, mitochondrial dysfunction and chronic inflammation in TAC-induced HF.** (A) Chord diagrams showing differentially expressed genes corresponding to selected regulated pathways in TAC + JV101 versus TAC + Vector comparisons. (B and C) Immunoblotting analysis of multiple inflammatory cytokines in heart of TAC-induced heart failure mice (n = 3 per group). (D) Nuclear lysate isolated from cardiomyocytes perfused from mice of TAC + Vector, TAC + JV101 and Sham group were immunoblotted for gH2AX, Histone H2AX, and 53BP1; Whole cell lysate was immunoblotted for p-p53(Ser15), p53, PGC1 a and TFAM. H3 and GAPDH were used as loading controls. (E–G) Representative transmission electron micrographs of left ventricular myocardium of the indicated study groups. Quantitative analyses of cristae number (F) and mitochondria density (G) in TAC hearts. (H) Quantification of mitochondrial DNA (mtDNA) copy numbers of cardiomyocytes perfused from mice of TAC + Vector, TAC + JV101 and Sham group (n = 6). (I) Intracellular ATP content of TAC + Vector, TAC + JV101 and Sham group (n = 6). Data are represented as mean ± SEM. ∗*P* < 0.05, ∗∗*P* < 0.01, ∗∗∗*P* < 0.001. Statistical differences among groups were analysed by one-way ANOVA followed by Tukey's multiple comparisons test.

Based on our proposed mechanism of action, we next wanted to verify whether JV101 functioned through telomeric reprotection. First, we evaluated the degree of expression levels of the shelterin complex. The results showed that JV101 did not rescue the decrease in shelterin gene *Trf2*, *Tin2*, *Tpp1*, and *Pot1a* mRNA expression levels as well as protein expression levels in isolated TAC cardiomyocytes (Supplementary Figure S3A–S3C). We next employed RNA-sequencing to analyse the cardiomyocytes that were isolated from TAC mice that were treated with or without JV101 as well as sham controls. The results revealed 26 significantly differentially expressed genes (DEGs) overlapping between various comparisons (Supplementary Figure S3D) and are represented as a heatmap (fold change >2 in log2 scale and adjusted p < 0.05; Supplementary Figure S3E). Gene function analysis revealed multiple significantly enriched biological processes, including inflammatory response, mitochondrial genome maintenance, and ATP metabolic process (Fig. 5A). Using Proteome Profiler Mouse XL Cytokine Array panel, we measured 111 different inflammatory cytokine protein levels in cardiac tissue from our JV101 treated animals. Of these, CCL6, CCL12, CXCL10, CXCL11, GDF-15, CXCL9, CXCL13, IL-1α, IL-1β, IL-33, MMP3, OPN, TNF-α, and PCSK9 inflammation-related cytokine proteins were significantly induced in TAC hearts compared to Sham controls that showed a clear reversal in the JV101 treated hearts (Fig. 5B and C).

In accord with the activation of the DNA damage response pathway, the transcript levels of p53 were increased in TAC cardiomyocytes but restored after JV101 treatment (Supplementary Figure S3F). PGC-1 α (PPARG coactivator 1α) is a master regulator of mitochondrial biogenesis and metabolism and have been shown to be repressed by p53 in the heart.^50^ Quantitative real-time polymerase chain reaction (RT-qPCR) analysis revealed that JV101 rescued the PGC-1α expression decrease after TAC (Supplementary Figure S3G). To confirm that p53 activation is directly due to uncapped telomeric ends, we performed 53BP1 immunofluorescence and telomeric Q-FISH colocalisation staining (Telomere dysfunction Induced Foci, TIF). In isolated TAC cardiomyocytes, TIF significantly increased, but JV101 rescued this change (Supplementary Figure S4A and S4B). In addition, immunoblotting of isolated cardiomyocytes showed an increase in γH2AX, 53BP1 and p53 ser15-phosphorylation protein levels, and this increase was reversed by JV101 treatment (Fig. 5D). Meanwhile, we observed JV101 significantly restored PGC-1 α and mitochondrial transcription factor A (TFAM) protein expression levels (Fig. 5D and Supplementary Figure S4C). By transmission electron microscopy, we found that JV101 treatment rescued mitochondrial cross-sectional area and cristae content reduction after TAC (Fig. 5E–G). In AMCMs, JV101 treatment rescued mitochondrial amount (Mito-tracker Green) and cell ROS (Supplementary Figure S4D–S4F). Ultimately, mtDNA copy number (Fig. 5H) and ATP production (Fig. 5I) were rescued by JV101 treatment. To confirm if p53 is a key mediator in JV101 treatment of heart failure, we repeated *in vivo* experiments in a myocardial p53-deficient mouse model (Myh6^Cre^ x p53^flox/flox^; p53^CKO^). Compared to wild type TAC animals, p53^CKO^ TAC animals displayed a less severe heart function decline, and JV101 treatment did not reverse cardiac dysfunction (Supplementary Figure S5 and Supplementary Table S6).

**Fig. 6:**
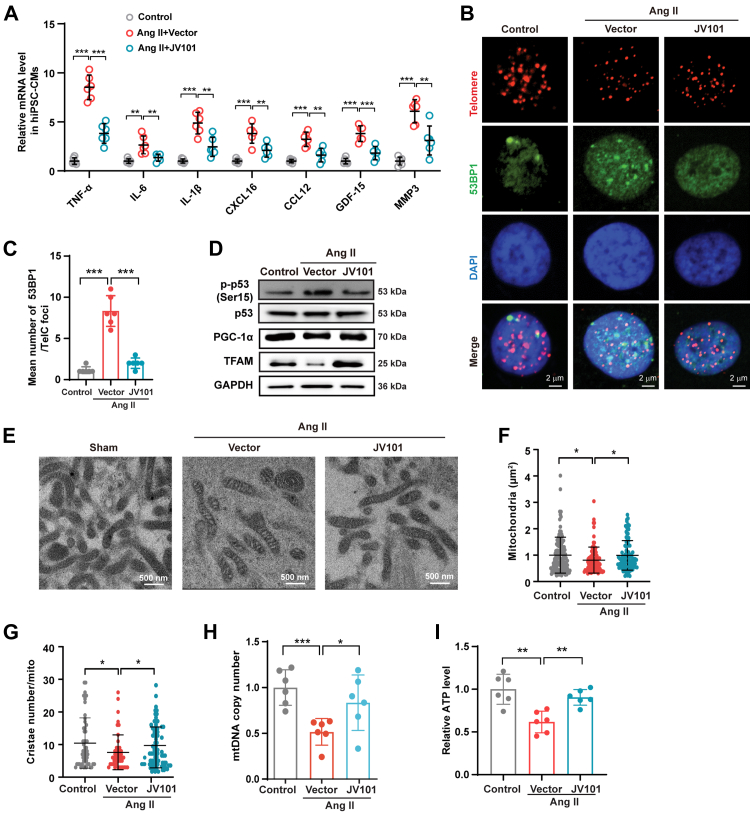
**JV101 blocked DDR-p53-TFAM axis and inflammation response in Ang II-induced hiPSC-CMs.** (A) Expression levels of inflammatory cytokines in hiPSC-CMs were determined by RT-qPCR (n = 6). (B) Representative micrographs of hiPSC-CMs displaying Telomere (red), 53BP1 (green) and DAPI (blue). (C) Quantification of 53BP1-telomere co-localisation (n = 6). (D) Cell lysate isolated from Ang II + Vector, Ang II + JV101 and control hiPSC-CMs were immunoblotted for p-p53(Ser15), p53, PGC-1a and TFAM. (E–G) Representative transmission electron micrographs of left ventricular myocardium of the indicated study groups. Quantitative analyses of cristae number (F) and mitochondria density (G) in hiPSC-CMs. (H) Quantification of mitochondrial DNA (mtDNA) copy numbers in different groups (n = 6). (I) Intracellular ATP content of different groups (n = 6). Data are represented as mean ± SEM. ∗*P* < 0.05, ∗∗*P* < 0.01, ∗∗∗*P* < 0.001. Statistical differences among groups were analysed by one-way ANOVA followed by Tukey's multiple comparisons test.

Fibrosis is regarded as a hallmark of late-stage heart failure triggered by myocardial loss and inflammation.^51^ JV101 overexpression significantly reduced inflammatory cytokine levels in Ang II treated hiPSC-CMs (Fig. 6A). In absence of p53, inflammatory cytokines (Supplementary Figure S6A) and ROS levels (Supplementary Figure S6B and S6C) were significantly decreased in cardiomyocytes isolated from p53^CKO^ TAC mice compared to TAC hearts. JV101 administration showed no further inhibition on inflammatory cytokines and ROS (Supplementary Figure S6A–S6C). We performed HA immunofluorescence staining coupled with telomeric FISH and confirmed nuclear localisation of modhTERT and its co-localisation with telomeric ends (Supplementary Figure S7A). Compared to Ang II treated hiPSC-CMs, nuclear HA-modhTERT expression prevented TIF accumulation (Fig. 6B and C), blocked p53 activation (Ser15-phosphorylation), rescued PGC-1 α and TFAM protein expression (Fig. 6D and Supplementary Figure S7B), preserved mitochondrial ultrastructure (Fig. 6E–G), restored mtDNA copy number (Fig. 6H), ameliorated ATP production (Fig. 6I), and reduced ROS levels (Supplementary Figure S7C), but did not reverse myocardial senescence (Supplementary Figure S7D and S7E). Together, these results demonstrate that JV101 treatment restores cardiac function *in vivo* and *in vitro* through telomere protection. Molecularly, modhTERT binds to deprotected telomeric ends and blocks DNA damage activation, reverts mitochondrial dysfunction, and dampens chronic inflammation via the telomere-p53-mitochondria signalling pathways (Supplementary Figure S8).

## Discussion

In this study, we found that myocardial telomeres correlated positively with left ventricular ejection fraction (LVEF%) in cynomolgus monkeys and HF patients. To test if telomere re-protection is sufficient to restore heart function, we designed an AAV9 to overexpress catalytically inactive and nuclear-localised telomerase protein (modhTERT^Y707F, D868A^) as a gene therapy (JV101) for heart failure. The mechanism of action entails modhTERT^Y707F, D868A^ binding to uncapped telomeric ends to block DNA damage response and restore mitochondrial biogenesis. Here we demonstrate the biodistribution and efficacy of JV101 in TAC heart failure murine models. One dose of JV101 (4 × 10^12^vg/kg) sufficiently restored cardiac function and cardiac fibrosis independent of telomere lengthening in TAC animals. *In vitro*, hiPSC-CMs hypertrophy were rescued after AAV9-modhTERT treatment. Molecularly, modhTERT^Y707F, D868A^ silenced telomeric DNA damage activation, restored mitochondrial biogenesis and ultrastructure, restored mitochondrial respiration and ATP production, and reversed chronic inflammation. These preclinical studies demonstrate that targeting telomeres has considerable potential for heart failure gene therapy.

The complex process of telomere replication is regulated by multiple telomere binding proteins.^52^ Under physiological conditions, human telomeres are capped by the shelterin protein complex which consists of TRF1, TRF2, RAP1, TIN2, TPP1, POT1.^53^^,^^54^ JV101, although sufficient in telomere re-protection, failed to rescue shelterin protein expression in diseased cardiomyocytes. Importantly, TAC mice myocardial telomere length was maintained rather than extended JV101 treatment at 56 days. These results indicate that JV101 efficacy is likely to act through telomere protection (TIF reduction) rather than restoring telomere length and do not pose the potential risk of immortalising cells. Handfuls of literature have demonstrated that overexpression of TERT, can ameliorate coronary heart disease (CHD), myocardial ischemia/reperfusion (I/R) injury, HF, and doxorubicin cardiotoxicity.^24^^,^^25^^,^^27^^,^^32^ Here, we not only demonstrate the efficacy of JV101 against HF, but through our protein design, we eliminate the potential risk of telomerase activity which may be oncogenic^55^ and random insertion of telomeric repeats.^56^

Even though our data demonstrates that JV101 treatment confers cardiac protection and may serve as a potential gene therapeutic option for heart failure, there are several limitations in the mechanistic explanations of causality and in the relationship between inflammatory states and mitochondrial dysfunction. Furthermore, this study included patients with heart failure (NYHA functional class II–IV) who had dilated cardiomyopathy (DCM), coronary heart disease (CHD), and ischaemic cardiomyopathy (ICM). The telomere length in human failing hearts correlated negatively with NYHA II to IV classification, implying that telomere reprotection plays a crucial role in heart failure with various etiologies. Besides the TAC-induced mouse HF model, JV101 may exert myocardial protective effects in other HF conditions. Conducting large-scale clinical case studies to confirm the role of JV101 among patients with heart failure of diverse etiologies would be highly valuable and significant for future investigations.

Telomeres and mitochondria are intimately linked. Telomere attrition induces metabolic and mitochondrial compromise.^57^^,^^58^ Notably, the premature ageing phenotypes associated with mitochondrial dysfunction mirror those of telomerase-deficient mice, p53-hyperactivated mice,^59^ and PGC1 α/β-null mice.^60^ SA-β-gal staining revealed that JV101 had no reversed Ang II-induced hiPSC-CMs senescence. Our previous reports found dystrophic cardiomyopathy induced telomere shortening and p53 activation-mediated metabolic damage.^23^ Additionally, the complex relationship between telomerase activity and inflammation significantly impacts cardiovascular diseases, diabetes, neurodegenerative disorders, and certain cancers.^58^^,^^61^, ^62^, ^63^ Telomerase reactivation has been shown to suppress inflammation and promote regeneration in several murine models^64^^,^^65^; however, whether telomere uncapping is responsible for myocardial inflammatory response remains unresolved. We and others have previously demonstrated that cGAS-STING pathway is activated in heart failure models and its activation drives inflammation.^66^^,^^67^ Cytosolic p53 has been shown to participate in cGAS-STING activation^68^ but more work is warranted to elucidate the degree of p53-cGAS-STING contribution. In parallel, p53 has also been shown to stimulate the production of pro-inflammatory cytokines.^69^ Our p53^CKO^ TAC results suggest p53 can induce myocardial inflammation directly. Our results indicate that heart failure-induced telomere attrition communicates with mitochondria via the TIF-p53-TFAM signalling axis. This pathway is accompanied by the production of chronic inflammation and reactive oxygen species (ROS). Based on the treatment effects of JV101, we speculate that JV101 may have a beneficial therapeutic impact on all heart failures with a reduced ejection fraction, but more efficacy studies are warranted. In sum, telomeric re-protection by JV101 rescues TAC heart failure serves as a proof-of-concept for a pan heart failure gene therapy and further development toward clinical application is warranted.

## Contributors

Experiments were designed, executed and analysed by Y.Z., X.B., W.X., X.W., Q.Y., T.W., A.C.H.C., Y.Y.L., Y.W., C.S., W.H., and A.C.Y.C. Reagents and conceptual assistance were provided by M.W., E.A.A., M.L., and Y.W. The manuscript was written by Y.Z., X.B., and A.C.Y.C. with input from all authors. W.H. and A.C.Y.C. supervised the study and acquired funding. Y.Z., X.B., W.X., and A.C.Y.C. have accessed and verified the underlying data. All authors have read and approved the final version of the manuscript.

## Data sharing statement

The RNA-sequence data generated in this study have been deposited in the Gene Expression Omnibus (GEO) database. The related RNA-seq data were available from GEO dataset (GSE234867). Other data are available in this article and its Supplementary Files or from the corresponding author upon request.

## Declaration of interests

E.A.A. serves as a consultant for Apple, Cathay Capital, Foresite Capital, Genome Medical, Medical Excellence Capital, Novartis, SequenceBio, Third Rock Ventures, and Walt Disney Company. E.A.A. receives grant funding/contract from Analogue Devices, AstraZeneca, Bristol Myers Squibb Company, Google, Illumina, Inc., Oxford Nanopore Technologies, PacBio, Samsung, Takeda California, Inc., and Verily. E.A.A. holds equity of DeepCell, Personalis, and Silicon Valley Exercise Analytics. None of these companies had any input into the design, execution, analyses or writing of this manuscript.

Q.Y. and Y.W. are employees of Juvensis Therapeutics. A.C.Y.C. (founder) is CEO and equity holder of Juvensis Therapeutics. Y.Y.L. (co-founder) is equity holder of Juvensis Therapeutics. Juvensis Therapeutics sponsored all the GMP grade AAV9-modTERT viruses (JV101) for this study but did not have any input into the design, execution, analyses or writing of this manuscript.

Other authors declare no competing interests.

## Appendix A Supplementary data

Supplementary Table 6

Supplementary Excel

Supplementary Western Blots
