# Supplementary material for: Modified hTERT treatment ameliorates pressure overload-induced heart failure
Source: eBioMedicine. 2026 Mar 9;126:106203. doi: 10.1016/j.ebiom.2026.106203 (PMC12993239; doi:10.1016/j.ebiom.2026.106203)
Supplement: Supplementary Table 1 [file mmc1.docx]

Table S1. Information for hiPSC lines

| Classification | hiPSC | Age | Tissue | Sex | Source |
| --- | --- | --- | --- | --- | --- |
| Healthy | Con# | 16 | Urine mesenchymal | Male | University of Washington |
