# Supplementary material for: Modified hTERT treatment ameliorates pressure overload-induced heart failure
Source: eBioMedicine. 2026 Mar 9;126:106203. doi: 10.1016/j.ebiom.2026.106203 (PMC12993239; doi:10.1016/j.ebiom.2026.106203)
Supplement: Supplementary Table 2 [file mmc2.docx]

Table S2. Primers for RT-qPCR

| Species | Gene | Forward | Reverse | Species | Gene | Forward | Reverse |
| --- | --- | --- | --- | --- | --- | --- | --- |
| Human | TERT | GCGGAAGACAGTGGTGAACT | TTCAGGATGGAGTAGCAGAGG | Mouse | Gapdh | ACTCCACTCACGGCAAATTCA | GGCCTCACCCCATTTGATG |
| Human | GAPDH | GTCTTCACCACCATGGAGAAGG | GCCTGCTTCACCACCTTCTTGA | Mouse | Anp | TTCGGGGGTAGGATTGACAG | CACACCACAAGGGCTTAGGA |
| Human | TNF-α | GCTGCACTTTGGAGTGATCG | GCTTGAGGGTTTGCTACAACA | Mouse | Bnp | TGTTTCTGCTTTTCCTTTATCTG | TCTTTTTGGGTGTTCTTTTGTGA |
| Human | IL-6 | CCGGGAACGAAAGAGAAGCTC | ACCGAAGGCGCTTGTGGAG | Mouse | β-Mhc | AGGCAAAGAAAGGCTCATCC | TGGAGCGCAAGTTTGTCATA |
| Human | IL-1β | CAGGCTGCTCTGGGATTCTC | GTCCTGGAAGGAGCACTTCAT | Mouse | p53 | TCCGAAGACTGGATGACTGC | GATCGTCCATGCAGTGAGGT |
| Human | CXCL16 | CTCTCCAGATCTGCCGGTTC | TGTCAGGGGTCAGTCTCCTT | Mouse | Pgc1a | CCTCGTTTTATTTGCATCCAGAGC | ACCAACCAGAGCAGCACACT |
| Human | CCL12 | CATGCTGAAGCTCACACCCT | GTCCCTGAGGGCTGAAAGTG | Mouse | Trf1 | ACAGCGCCGAGGCTATTATT | GCATCAAGGGCCTTTCCAGA |
| Human | GDF-15 | ATACTCACGCCAGAAGTGCGG | CTTGCAAGGCTGAGCTGACG | Mouse | Tpp1 | CTACTGGGTGGTCAGCAACA | CAGCCGTGGGTTACATCAAAG |
| Human | MMP3 | CACAGACCTGACTCGGTTCC | TCAGGGGGAGGTCCATAGAG | Mouse | Tin2 | AGTCAAGAAACCAGGATCTTCC | CCTTTGACATGGGCAGTTGAG |
| Human | TERT-Taqman | GAGGGTGAAGGCACTGTTCA | CCGTCACATCCACCTTGACA | Mouse | Pot1a | TCACCAGCAGGAATGAAAGATTTA | TGAAACCAAAGACATTGTTTGATGA |
| Human | TERT-probe | CTCTGTGCTGGGCCTGGACGATATC |  | Mouse | Pot1b | CTACCTCAAGGATTCTGAGAAGTT | CACCTTTGAAATGCTGCCTACC |
| Human | MTRNR2-mtDNA | CGAAAGGACAAGAGAAATAAGG |  | Mouse | Rap1 | GGGTCAGGAGCACAAGTACC | TCTTGTTCTGTGGCTCTCCG |
| Human | MTTL1-mtDNA |  | CTGTAAAGTTTTAAGTTTTATGCG | Mouse | Trf2 | GAGCCTCCAAGCAGACAGC | TGTTTGTGGGCTGGTGATGA |
| Human | β-GLOBI-mtDNA | CAACTTCATCCACGTTCACC | GAAGAGCCAAGGACAGGTAC | Mouse | Cytb-mtDNA | GCTTTCCACTTCATCTTACCATTTA | TGTTGGGTTGTTTGATCCTG |
|  |  |  |  | Mouse | β-Actin | GGAAAAGAGCCTCAGGGCAT | GAAGAGCTATGAGCTGCCTGA |
|  |  |  |  | Mouse  Mouse  Mouse  Mouse  Mouse | TNF-α  IL-1β  CCL12  GDF-15  MMP3 | ACCCTCACACTCACAAACCA  TGCCACCTTTTGACAGTGATG  TATTGGCTGGACCAGATGCGG  ATCTGAGCCTGAGACGAACT  CAGTCCCTCTATGGAACTCCC | ACAAGGTACAACCCATCGGC  TGATGTGCTGCTGCGAGATT  ACACTGGCTGCTTGTGATTCT  CCTCAGTATCCCCAGACCAG  AGGGTGCTGACTGCATCAAA |
