# Supplementary material for: Modified hTERT treatment ameliorates pressure overload-induced heart failure
Source: eBioMedicine. 2026 Mar 9;126:106203. doi: 10.1016/j.ebiom.2026.106203 (PMC12993239; doi:10.1016/j.ebiom.2026.106203)
Supplement: Supplementary Table 3 [file mmc3.docx]

Table S3. Temporal monitoring of cardiac function in Sham, TAC+Vector, and TAC treated with JV101 mice

|  |  | Time(week) | | | | | | | | | |
| --- | --- | --- | --- | --- | --- | --- | --- | --- | --- | --- | --- |
|  | Group | 0 | 7 | 14 | 21 | 28 | 35 | 42 | 49 | 56 |  |
| Diameter;s | Sham | 1.58±0.21 | 1.75±0.59 | 1.55±0.3 | 1.73±0.37 | 1.71±0.48 | 1.7±0.31 | 1.8±0.62 | 1.84±0.21 | 1.76±0.15 |  |
|  | TAC | 1.67±0.3 | 2.19±0.17* | 2.48±0.17*** | 2.39±0.27*** | 2.62±0.23*** | 2.77±0.24*** | 3.08±0.16*** | 3.2±0.25*** | 3.5±0.3*** |  |
|  | TAC+JV101 | 1.74±0.45 | 2.32±0.06 | 2.4±0.18### | 2.63±0.3### | 2.44±0.13 | 2.77±0.28 | 2.43±0.22### | 2.43±0.36### | 2.46±0.33### |  |
| Diameter;d | Sham | 3.13±0.31 | 3.49±0.41 | 3.34±0.17 | 3.26±0.4 | 3.36±0.35 | 3.31±0.37 | 3.59±0.57 | 3.41±0.17 | 3.4±0.22 |  |
|  | TAC | 3.35±0.29 | 3.33±0.25 | 3.63±0.28*** | 3.55±0.25** | 3.69±0.36 | 3.77±0.25* | 4±0.16 | 4.1±0.26*** | 4.22±0.35*** |  |
|  | TAC+JV101 | 3.35±0.36 | 3.73±0.12 | 3.62±0.38 | 3.72±0.3 | 3.7±0.16 | 3.89±0.26 | 3.5±0.28## | 3.59±0.45# | 3.7±0.28## |  |
| Volume;s | Sham | 7.15±2.41 | 10.66±7.91 | 7±3.31 | 9.4±5.11 | 9.57±6.17 | 8.84±3.79 | 11.6±8.19 | 10.53±3.05 | 9.37±2.14 |  |
|  | TAC | 8.56±4 | 16.24±3.24 | 22.16±3.69* | 20.43±5.78 | 25.41±5.36*** | 29.14±6.11*** | 37.53±4.52*** | 41.36±7.77*** | 51.49±11.15*** |  |
|  | TAC+JV101 | 9.91±5.02 | 18.51±1.28 | 20.33±3.82 | 25.94±6.91 | 21.24±2.95 | 29.27±7.2 | 21.05±5.18### | 21.62±8.1### | 22.06±7.92### |  |
| Volume;d | Sham | 39.45±9.33 | 51.44±14.42 | 45.46±5.66 | 43.89±12.1 | 46.65±11.73 | 45.23±11.91 | 55.88±22.24 | 48.11±5.92 | 47.73±7.36 |  |
|  | TAC | 46.31±10.01 | 45.59±8.17 | 56.05±10.49*** | 52.9±8.53** | 58.59±13.03 | 61.01±9.53* | 70.21±6.58 | 74.6±11.16*** | 80.14±16.19*** |  |
|  | TAC+JV101 | 46.74±11.55 | 59.41±4.59 | 56.11±14.02 | 59.27±11.23 | 58.36±6 | 65.83±10.7 | 51.2±10.67## | 55.25±16.27# | 58.52±10.91## |  |
| Stroke Volume | Sham | 32.29±7.53 | 40.78±6.89 | 38.46±5.22 | 34.49±9.37 | 37.08±6.68 | 36.39±8.98 | 44.28±15.36 | 37.59±3.25 | 38.37±6.41 |  |
|  | TAC | 37.76±8.22 | 29.35±5.47** | 33.89±7.75* | 32.47±4.56 | 33.18±9.42 | 31.87±3.84 | 32.68±3.18 | 33.24±4.79 | 28.65±6.26* |  |
|  | TAC+JV101 | 36.83±8.24 | 40.9±3.43 | 35.78±10.75 | 33.33±5.36 | 37.12±5.6 | 36.56±6.2 | 30.15±6.19 | 33.63±10.99 | 36.45±5.25# |  |
| EF | Sham | 81.95±3.88 | 81.55±10.14 | 84.66±6.63 | 79.03±7.83 | 80.94±9.07 | 80.83±5.52 | 80.62±8.98 | 78.46±3.95 | 80.27±3.69 |  |
|  | TAC | 81.62±7.16 | 64.34±3.17*** | 60.19±4.07 | 61.87±5.98 | 56.12±5.6*** | 52.57±3.38*** | 46.62±2.83*** | 44.76±3.86*** | 35.74±3.96*** |  |
|  | TAC+JV101 | 79.62±8.36 | 68.82±0.87 | 63.04±4.8 | 56.75±5.25 | 63.45±4.97# | 55.75±7.01 | 58.97±4.09### | 60.82±8.11### | 62.96±6.94### |  |
| FS | Sham | 49.65±4.13 | 51.05±11.36 | 53.67±8.44 | 47.21±8.01 | 49.82±9.98 | 48.89±6.41 | 50.52±13.02 | 46.25±3.73 | 48.06±3.79 |  |
|  | TAC | 50.25±7.73 | 34.19±2.41*** | 31.51±2.94*** | 32.67±4.12*** | 28.84±3.97*** | 26.51±2.05*** | 22.99±1.64*** | 21.95±2.19*** | 16.96±2.14*** |  |
|  | TAC+JV101 | 48.67±10.63 | 37.92±0.71 | 33.53±3.55 | 29.24±3.36 | 33.9±3.54# | 28.84±4.57 | 30.54±2.82### | 32.13±5.79### | 33.66±4.91### |  |
| Cardiac Output | Sham | 14.83±3.34 | 18.09±3.25 | 18.64±2.6 | 16.15±4.67 | 15.34±2.8 | 16.26±5.09 | 17.65±3.43 | 17.18±1.74 | 16.56±1.73 |  |
|  | TAC | 18.31±4.41 | 13.81±3.01* | 15.41±4.33*** | 14.54±2.57 | 14.57±3.02 | 14.24±2.88 | 14.8±2.07 | 14.46±2.02* | 12.99±3.21* |  |
|  | TAC+JV101 | 17.7±4.01 | 18.76±2.56 | 16.67±5.23 | 14.53±2.28 | 15.8±3.17 | 17.33±3.49 | 14.62±3.03 | 14.87±4.97 | 17.12±2.6# |  |
| LV Mass | Sham | 130.07±34.62 | 135.07±16.62 | 117.57±18.03 | 129.64±24.53 | 132.46±33.43 | 130.95±9.55 | 143.25±71.86 | 118.75±24 | 121.9±13.72 |  |
|  | TAC | 125.24±24.94 | 119.85±28.58 | 117.04±27.8 | 124.04±34.63 | 121.64±19.84 | 144.62±59.18 | 127.6±23.67 | 148.46±28.01 | 169.91±48.13* |  |
|  | TAC+JV101 | 139.47±26.84 | 110.48±18.62 | 118.56±24.28 | 133.37±29.14 | 131.28±28.4 | 130.28±26.92 | 141.93±35.24 | 113.67±33.67# | 168.03±31.3 |  |
| LV Mass Cor | Sham | 104.05±27.7 | 108.05±13.3 | 94.05±14.42 | 103.71±19.63 | 105.97±26.74 | 104.76±7.64 | 114.6±57.48 | 95±19.2 | 97.52±10.98 |  |
|  | TAC | 100.2±19.95 | 95.88±22.86 | 93.63±22.24 | 99.23±27.7 | 97.31±15.87 | 115.7±47.34 | 102.08±18.94 | 118.77±22.4 | 135.93±38.51 |  |
|  | TAC+JV101 | 111.58±21.47 | 88.38±14.9 | 94.84±19.42 | 106.69±23.31 | 105.02±22.72 | 104.23±21.53 | 113.54±28.19 | 90.93±26.93# | 134.43±25.04 |  |
| LVAW; s | Sham | 1.77±0.26 | 1.74±0.27 | 1.79±0.1 | 1.76±0.28 | 1.64±0.25 | 1.7±0.26 | 1.67±0.27 | 1.59±0.26 | 1.64±0.23 |  |
|  | TAC | 1.77±0.28 | 1.38±0.17** | 1.32±0.14 | 1.32±0.19 | 1.24±0.08* | 1.46±0.4 | 1.26±0.16** | 1.29±0.17* | 1.33±0.31 |  |
|  | TAC+JV101 | 1.82±0.31 | 1.38±0.15 | 1.38±0.13 | 1.37±0.24 | 1.36±0.2 | 1.3±0.2 | 1.49±0.25# | 1.35±0.2 | 1.55±0.22 |  |
| LVAW;d | Sham | 1.11±0.24 | 1.07±0.11 | 1.09±0.07 | 1.12±0.18 | 0.98±0.18 | 1.05±0.12 | 0.95±0.04 | 0.95±0.16 | 0.99±0.11 |  |
|  | TAC | 1.07±0.2 | 0.93±0.08 | 0.89±0.05*** | 0.91±0.16** | 0.91±0.09 | 1.07±0.33 | 0.91±0.16 | 0.91±0.14 | 1.02±0.26 |  |
|  | TAC+JV101 | 1.09±0.15 | 0.89±0.11 | 0.95±0.14 | 0.98±0.17 | 0.9±0.13 | 0.91±0.16 | 1.05±0.23 | 0.9±0.12 | 1.05±0.19 |  |
| LVPW; s | Sham | 1.69±0.32 | 1.64±0.16 | 1.6±0.31 | 1.62±0.31 | 1.74±0.24 | 1.69±0.23 | 1.74±0.47 | 1.55±0.22 | 1.61±0.2 |  |
|  | TAC | 1.57±0.28 | 1.47±0.32 | 1.23±0.18*** | 1.34±0.36* | 1.22±0.15** | 1.15±0.28** | 1.05±0.19** | 1.14±0.17** | 1.08±0.15*** |  |
|  | TAC+JV101 | 1.65±0.35 | 1.29±0.11 | 1.25±0.26 | 1.23±0.23 | 1.35±0.26 | 1.16±0.18 | 1.39±0.3# | 1.16±0.24 | 1.59±0.15### |  |
| LVPW;d | Sham | 1.11±0.32 | 0.99±0.11 | 0.87±0.23 | 1.03±0.3 | 1.12±0.19 | 1.08±0.14 | 1.05±0.28 | 0.97±0.21 | 0.98±0.13 |  |
|  | TAC | 0.96±0.18 | 1.05±0.34* | 0.88±0.19* | 0.98±0.34 | 0.9±0.22 | 0.89±0.26 | 0.78±0.17* | 0.93±0.14 | 0.92±0.15 |  |
|  | TAC+JV101 | 1.1±0.29 | 0.76±0.09 | 0.85±0.21 | 0.93±0.21 | 1.71±0.48 | 0.86±0.16 | 1.06±0.28# | 0.84±0.17 | 1.19±0.21# |  |
| *p＜0.05, **p＜0.01, ***p＜0.001 for TAC+Vector compared with Sham; #p＜0.05, ##p＜0.01, ###p＜0.001 for TAC+JV101 compared with TAC+Vector | | | | | | | | | | |  |
