# Supplementary material for: Modified hTERT treatment ameliorates pressure overload-induced heart failure
Source: eBioMedicine. 2026 Mar 9;126:106203. doi: 10.1016/j.ebiom.2026.106203 (PMC12993239; doi:10.1016/j.ebiom.2026.106203)
Supplement: Supplementary Table 4 [file mmc4.docx]

Table S4. Routine blood tests in Sham, TAC, and TAC +JV101 mice

|  | Sham | TAC+Vector | TAC+JV101 |
| --- | --- | --- | --- |
| WBC(K/uL) | 2.67±1.3 | 3.92±1.39 | 3.69±2.54 |
| NEUT#(K/uL) | 0.52±0.24 | 0.72±0.2 | 0.93±0.9 |
| LYMPH#(K/uL) | 2.09±1.13 | 2.95±1.08 | 2.58±1.81 |
| MONO#(K/uL) | 0.01±0.01 | 0.03±0.03 | 0.01±0.02 |
| EO#(K/uL) | 0.05±0.03 | 0.22±0.37 | 0.16±0.21 |
| BASO#(K/uL) | 0±0.01 | 0±0.01 | 0.01±0.01 |
| NEUT% (%) | 21.42±7.86 | 19±3.24 | 24.18±12.63 |
| LYMPH% (%) | 75.8±8.13 | 75.66±8.03 | 71.44±10.03 |
| MONO% (%) | 0.3±0.28 | 0.56±0.57 | 0.28±0.29 |
| EO% (%) | 2.37±2.24 | 4.64±6.38 | 3.96±3.04 |
| BASO% (%) | 0.12±0.18 | 0.14±0.31 | 0.14±0.19 |
| RBC(M/uL) | 8.97±4 | 11.29±0.37 | 9.33±3.9 |
| HGB(g/dL) | 12.46±5.54 | 15.34±0.44 | 12.75±5.23 |
| HCT (%) | 43.57±19.38 | 54.54±1.65 | 44.68±19.17 |
| MCV | 41.64±18.38 | 48.3±0.66 | 46.9±2.82 |
| MCH | 11.9±5.25 | 13.58±0.16 | 13.88±0.66 |
| MCHC(g/dL) | 28.58±0.26 | 28.12±0.15 | 29.75±3.46 |
| PLT (10^9^ /L) | 720.43±424.69 | 887±158.59 | 742.5±443.99 |
| RET (10^12^ /L) | 15.37±1.13 | 15.2±0.25 | 15.3±0.38 |
| RET% (%) | 3.62±0.76 | 3.06±0.58 | 3.05±0.58 |
| WBC, white blood cell count; NEUT, Neutrophil granulocyte; LYMPH, lymphocyte; MONO, monocyte; EO, eosinophil; BASO, basophil; RBC, red blood cell; HGB, hemoglobin; HCT, hematocrit; MCV, mean corpusular volume; MCH, mean corpusular hemoglobin; MCHC, mean corpusular hemoglobin concerntration; PLT, platelet count/blood platelet count. *p＜0.05, **p＜0.01, ***p＜0.001 for Ang TAC+Vector compared with Sham; #p＜0.05, ##p＜0.01, ###p＜0.001 for TAC+JV101 compared with TAC +Vector. | | | |
