# Supplementary material for: Modified hTERT treatment ameliorates pressure overload-induced heart failure
Source: eBioMedicine. 2026 Mar 9;126:106203. doi: 10.1016/j.ebiom.2026.106203 (PMC12993239; doi:10.1016/j.ebiom.2026.106203)
Supplement: Supplementary Table 5 [file mmc5.docx]

Table S5. Blood biochemical tests in Sham, TAC, and TAC+JV101 mice

|  | Sham | TAC+Vector | TAC+JV101 |
| --- | --- | --- | --- |
| ALTL(U/L) | 9.76±1.05 | 8.03±1.91 | 9.58±0.73 |
| ASTL(U/L) | 28.5±3.13 | 33.05±4.61 | 31.98±3.73 |
| LDLC3(mmol/L) | 0.07±0.02 | 0.09±0.03 | 0.09±0.01 |
| TRIGL (mmol/L) | 0.32±0.09 | 0.28±0.03 | 0.3±0.14 |
| CHO2I(mmol/L) | 0.65±0.1 | 0.69±0.07 | 0.68±0.04 |
| HDLC4(mmol/L) | 0.52±0.08 | 0.55±0.06 | 0.53±0.03 |
| CREA2(umol/L) | 2.13±0.96 | 2.5±0.94 | 4.28±1.15 |
| UREAL (mmol/L) | 2.05±0.3 | 2.45±0.39 | 2.54±0.44 |
| UA2(umol/L) | 33.01±4.87 | 46±9.65* | 56±5.75 |
| CK2(U/L) | 341.29±62.41 | 431.17±67.28* | 313.28±158.66 |
| CKMB2(U/L) | 64.26±5.72 | 69.3±10.97 | 84.38±18.98 |
| LDH(U/L) | 126.86±17.79 | 127.83±51.21 | 185.33±34.85# |
| ALTL, Alanine transaminase; ASTL, Aspartate transaminase; LDLC3, Low density lipoprotein cholesterol; TRIGL, Triglycerides; CHO2I, cholesterol; HDLC, High density lipoprotein cholesterol; CREA2, creatinine; UREAL, urea nitrogen; UA2, uric acid; CK2, casein kinase 2; CKMB2, creatine kinase isoenzyme; LDH, lactic dehydrogenase. *p＜0.05, **p＜0.01, ***p＜0.001 for TAC+Vector compared with Sham; #p＜0.05, ##p＜0.01, ###p＜0.001 for TAC+JV101 compared with TAC +Vector. | | | |
