# Supplementary material for: Modified hTERT treatment ameliorates pressure overload-induced heart failure
Source: eBioMedicine. 2026 Mar 9;126:106203. doi: 10.1016/j.ebiom.2026.106203 (PMC12993239; doi:10.1016/j.ebiom.2026.106203)
Supplement: Supplementary Table 6 [file mmc6.docx]

Table S6. Temporal monitoring of cardiac function in p53^CKO^ Sham, p53^CKO^ TAC+Vector, and p53^CKO^ TAC + JV101 mice

|  | Time (week) | | | |
| --- | --- | --- | --- | --- |
|  | Group | 0 | 2 | 8 |
| Diameter; s | p53^CKO^ Sham | 1.40±0.35 | 1.50±0.35 | 1.76±0.35 |
|  | p53^CKO^ TAC +Vector | 1.51±0.46 | 2.52±0.17*** | 2.78±0.26*** |
|  | p53^CKO^ TAC + JV101 | 1.61±0.40 | 2.66±0.21 | 2.60±0.33 |
| Diameter; d | p53^CKO^ Sham | 2.81±0.36 | 3.16±0.43 | 3.34±0.34 |
|  | p53^CKO^ TAC +Vector | 2.97±0.41 | 3.58±0.14* | 3.73±0.27* |
|  | p53^CKO^ TAC + JV101 | 3.08±0.33 | 3.76±0.26 | 3.67±0.43 |
| Volume; s | p53^CKO^ Sham | 5.62±3.17 | 6.70±4.00 | 9.78±5.22 |
|  | p53^CKO^ TAC +Vector | 7.24±4.01 | 22.89±3.91*** | 29.35±6.49*** |
|  | p53^CKO^ TAC + JV101 | 8.04±4.51 | 26.25±4.76 | 25.08±7.80 |
| Volume; d | p53^CKO^ Sham | 30.39±9.45 | 40.66±13.14 | 46.19±10.95 |
|  | p53^CKO^ TAC +Vector | 35.06±10.45 | 53.83±5.17* | 59.79±10.67* |
|  | p53^CKO^ TAC + JV101 | 37.06±9.84 | 60.78±9.49 | 58.21±16.54 |
| Stroke Volume | p53^CKO^ Sham | 24.77±7.14 | 33.96±9.43 | 36.41±6.56 |
|  | p53^CKO^ TAC +Vector | 27.82±6.90 | 30.94±3.10 | 30.44±5.81 |
|  | p53^CKO^ TAC + JV101 | 29.92±5.94 | 34.53±5.10 | 33.13±9.56 |
| EF | p53^CKO^ Sham | 82.43±8.05 | 84.55±4.87 | 79.78±6.15 |
|  | p53^CKO^ TAC +Vector | 81.25±8.29 | 57.60±4.69*** | 51.15±5.70*** |
|  | p53^CKO^ TAC + JV101 | 80.05±7.69 | 56.08±2.71 | 57.03±5.48 |
| FS | p53^CKO^ Sham | 50.71±8.48 | 52.83±5.46 | 47.68±5.70 |
|  | p53^CKO^ TAC +Vector | 50.22±11.09 | 29.71±3.19*** | 25.67±3.48*** |
|  | p53^CKO^ TAC + JV101 | 48.38±8.95 | 29.36±1.68 | 29.38±3.58 |
| Cardiac Output | p53^CKO^ Sham | 10.86±2.76 | 16.47±4.81 | 17.60±3.79 |
|  | p53^CKO^ TAC +Vector | 13.42±3.78 | 13.74±2.03 | 13.65±3.53 |
|  | p53^CKO^ TAC + JV101 | 13.72±2.33 | 16.78±2.09# | 13.65±4.77 |
| LV Mass | p53^CKO^ Sham | 120.80±24.87 | 120.70±30.29 | 110.30±22.56 |
|  | p53^CKO^ TAC +Vector | 112.50±30.63 | 111.90±21.13 | 98.55±22.32 |
|  | p53^CKO^ TAC + JV101 | 121.40±26.78 | 138.20±19.39# | 106.80±19.36 |
| LV Mass Cor | p53^CKO^ Sham | 96.62±19.89 | 96.57±24.23 | 88.22±18.05 |
|  | p53^CKO^ TAC +Vector | 90.01±24.50 | 89.48±16.90 | 78.84±17.85 |
|  | p53^CKO^ TAC + JV101 | 97.16±21.42 | 110.50±15.51# | 85.45±15.49 |
| LVAW; s | p53^CKO^ Sham | 1.78±0.27 | 1.65±0.26 | 1.70±0.24 |
|  | p53^CKO^ TAC +Vector | 1.61±0.21 | 1.36±0.18* | 1.10±0.20*** |
|  | p53^CKO^ TAC + JV101 | 1.69±0.16 | 1.38±0.15 | 1.24±0.17 |
| LVAW; d | p53^CKO^ Sham | 1.12±0.20 | 1.10±0.09 | 0.99±0.19 |
|  | p53^CKO^ TAC +Vector | 1.05±0.21 | 0.95±0.19 | 0.76±0.20 |
|  | p53^CKO^ TAC + JV101 | 1.04±0.15 | 0.94±0.13 | 0.860±.10 |
| LVPW; s | p53^CKO^ Sham | 1.69±0.23 | 1.58±0.23 | 1.33±0.16 |
|  | p53^CKO^ TAC +Vector | 1.58±0.29 | 1.09±0.18*** | 1.05±0.14** |
|  | p53^CKO^ TAC + JV101 | 1.54±0.18 | 1.26±0.26 | 1.09±0.15 |
| LVPW; d | p53^CKO^ Sham | 1.23±0.37 | 1.00±0.28 | 0.88±0.15 |
|  | p53^CKO^ TAC +Vector | 1.06±0.23 | 0.80±0.13 | 0.76±0.11 |
|  | p53^CKO^ TAC + JV101 | 1.12±0.21 | 0.99±0.23# | 0.79±0.17 |
| *p＜0.05, **p＜0.01, ***p＜0.001 for p53^CKO^ TAC +Vector compared with p53^CKO^ Sham; #p＜0.05, ##p＜0.01, ###p＜0.001 for p53^CKO^ TAC +JV101 compared with p53^CKO^ TAC +Vector | | | | |
