# Supplementary material for: Modified hTERT treatment ameliorates pressure overload-induced heart failure
Source: eBioMedicine. 2026 Mar 9;126:106203. doi: 10.1016/j.ebiom.2026.106203 (PMC12993239; doi:10.1016/j.ebiom.2026.106203)

**Red boxes in the uncropped blots indicate the cropped regions shown in the corresponding figures.**

**Uncropped blots for Figure 5D**


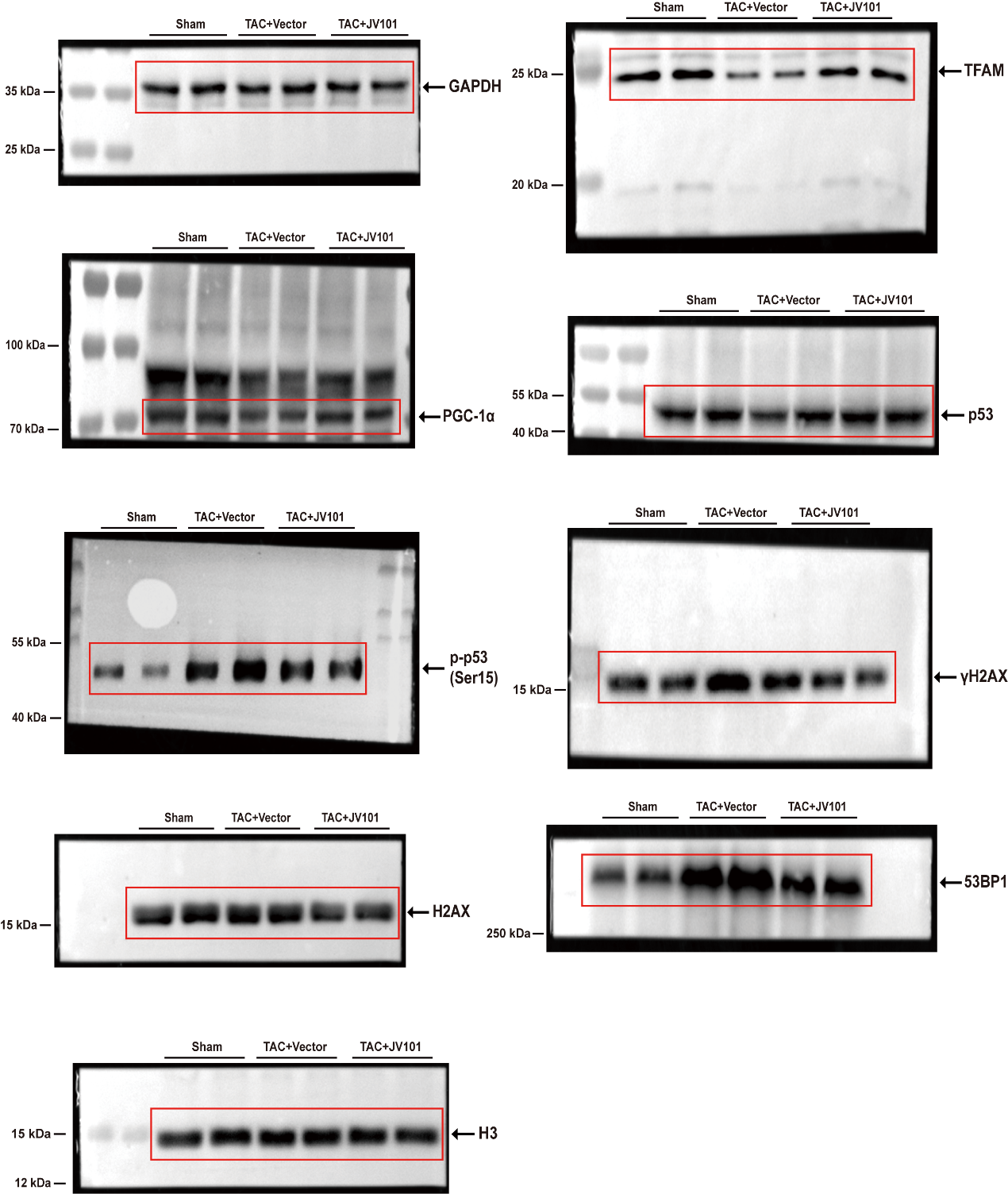


**Uncropped blots for Figure 6D**


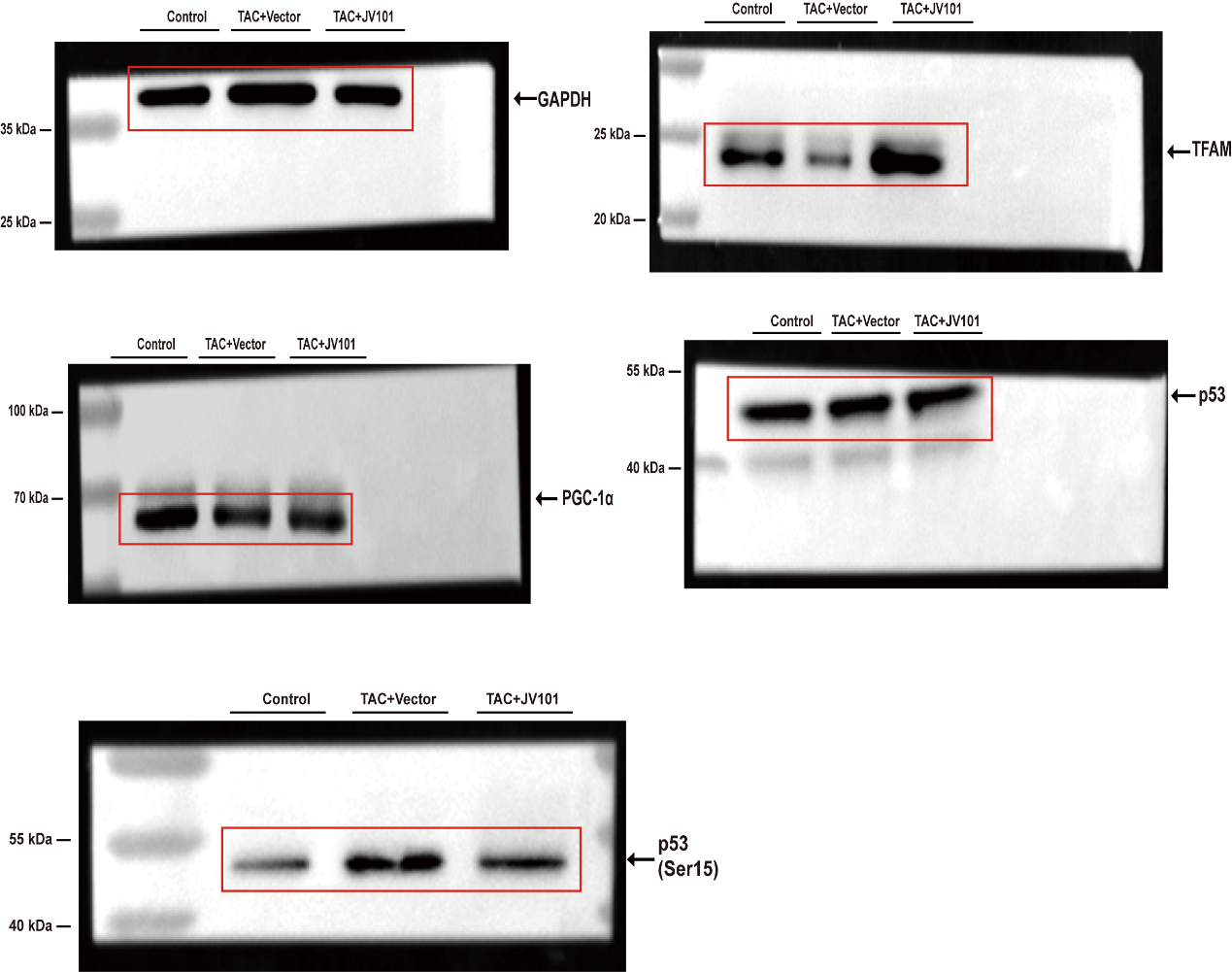


**Uncropped blots for Figure S3B**


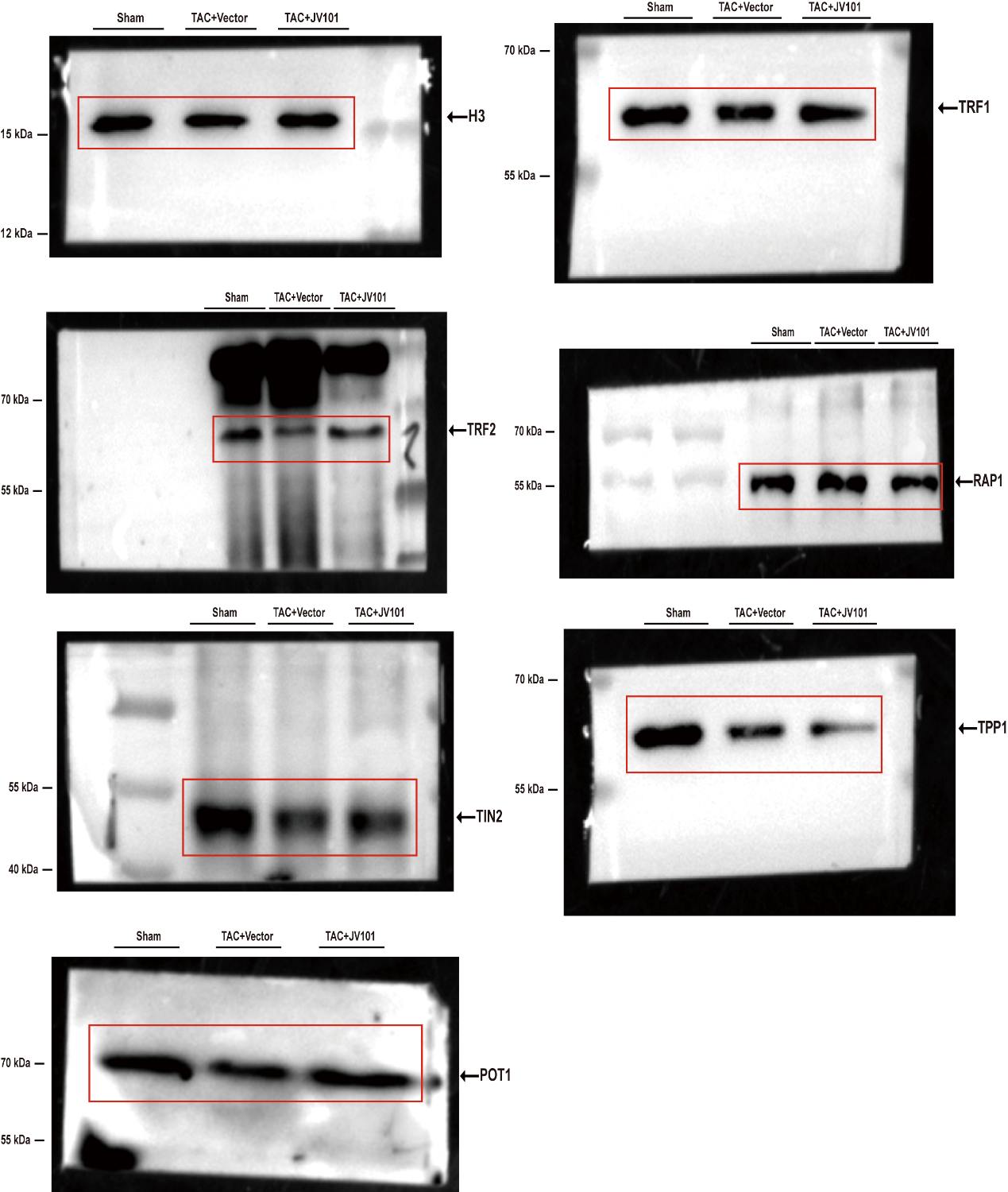

Supplement: Supplementary Western Blots [file mmc8.docx]
