# Supplementary material for: Modified hTERT treatment ameliorates pressure overload-induced heart failure
Source: eBioMedicine. 2026 Mar 9;126:106203. doi: 10.1016/j.ebiom.2026.106203 (PMC12993239; doi:10.1016/j.ebiom.2026.106203)
Supplement: Supplementary Figures [file mmc9.docx]

**Supplementary Figures and legends**

**Effects of modified hTERT treatment on pressure overload-induced heart failure**

Yinlong Zhao^1,2*^, Xiaolu Bao^1,2*^, Weiyao Xiong^1,2^, Xin Wan^1,2^, Qingying Yu^3^, Teng Wang^1,2^, Andrew C. H. Chang^1,2^, Yangyang Liu^3^, Yanqiu Wang^3^, Ching Shang^4^, Min Wu^5^, Euan A. Ashley^4^, Ming Lei^2^, Junfeng Zhang^1#^, Yueheng Wu^5#^, Wei Han^6#^, Alex C Y Chang^1,2#^

^1^Department of Cardiology, Ninth People’s Hospital, Shanghai Jiao Tong University School of Medicine, Shanghai 200125, China.

^2^Shanghai Institute of Precision Medicine, Ninth People’s Hospital, Shanghai Jiao Tong University School of Medicine, Shanghai 200125, China.

^3^Juvensis Therapeutics, Shanghai, China.

^4^ Department of Medicine, Division of Cardiology, Stanford University, Palo Alto,

CA, USA.

^5^Department of Cardiovascular Surgery, Guangdong Provincial Key Laboratory of South China Structural Heart Disease, Guangdong Cardiovascular Institute, Guangdong Provincial People’s Hospital (Guangdong Academy of Medical Sciences), Southern Medical University, Guangzhou, Guangdong, China.

^6^Department of Heart Failure, Heart Center, Shanghai East Hospital, School of Medicine, Tongji University, Shanghai 200120, China.

^*^These authors contributed equally.

^#^Correspondence should be addressed to: Alex C Y Chang ([alexchang@shsmu.edu.cn](mailto:alexchang@shsmu.edu.cn)); Wei Han ([dr.hanwei@foxmail.com](mailto:dr.hanwei@foxmail.com)); Yueheng Wu ([wuyueheng@gdph.org.cn](mailto:wuyueheng@gdph.org.cn)); Junfeng Zhang ([jfzhang_dr@163.com](mailto:jfzhang_dr@163.com))


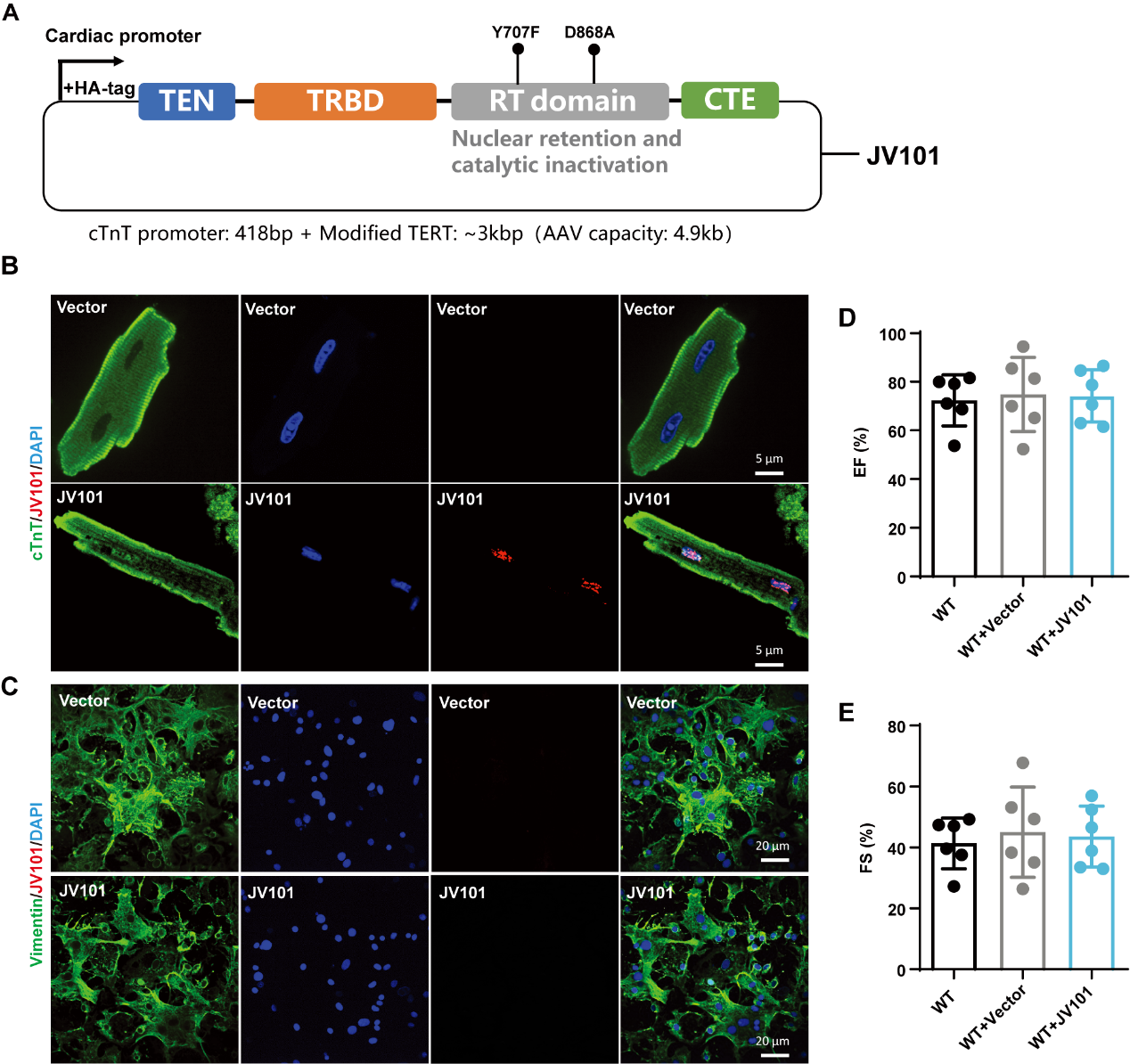


**Figure S1. Design and evaluation of HA-****modhTERT^Y707F, D868A^ in wildtype mice.** (A) Schematic representation of AAV9-cTNT-hTERT^Y707F, D868A^ (AAV9-modhTERT, JV101) gene design. Sequence analysis shows the two mutated sites, Y707 site of the A motif and D868 of the C motif in the RT domain. (B) Representative micrographs of Langendorff-isolated mouse cardiomyocytes stained for anti-HA (HA-modhTERTY707F, D868A), cardiac troponin (cTNT), and nuclei (DAPI). (C) Representative micrographs of Langendorff-isolated mouse fibroblast stained for anti-HA (HA-modhTERTY707F, D868A), Vimentin, and nuclei (DAPI). (D and E) Cardiac function evaluation by echocardiography in wildtype animals injected with JV101. Ejection fraction (EF%) (D) and fractional shortening (FS%) (E) are shown (n = 6). Data are mean ± SEM.


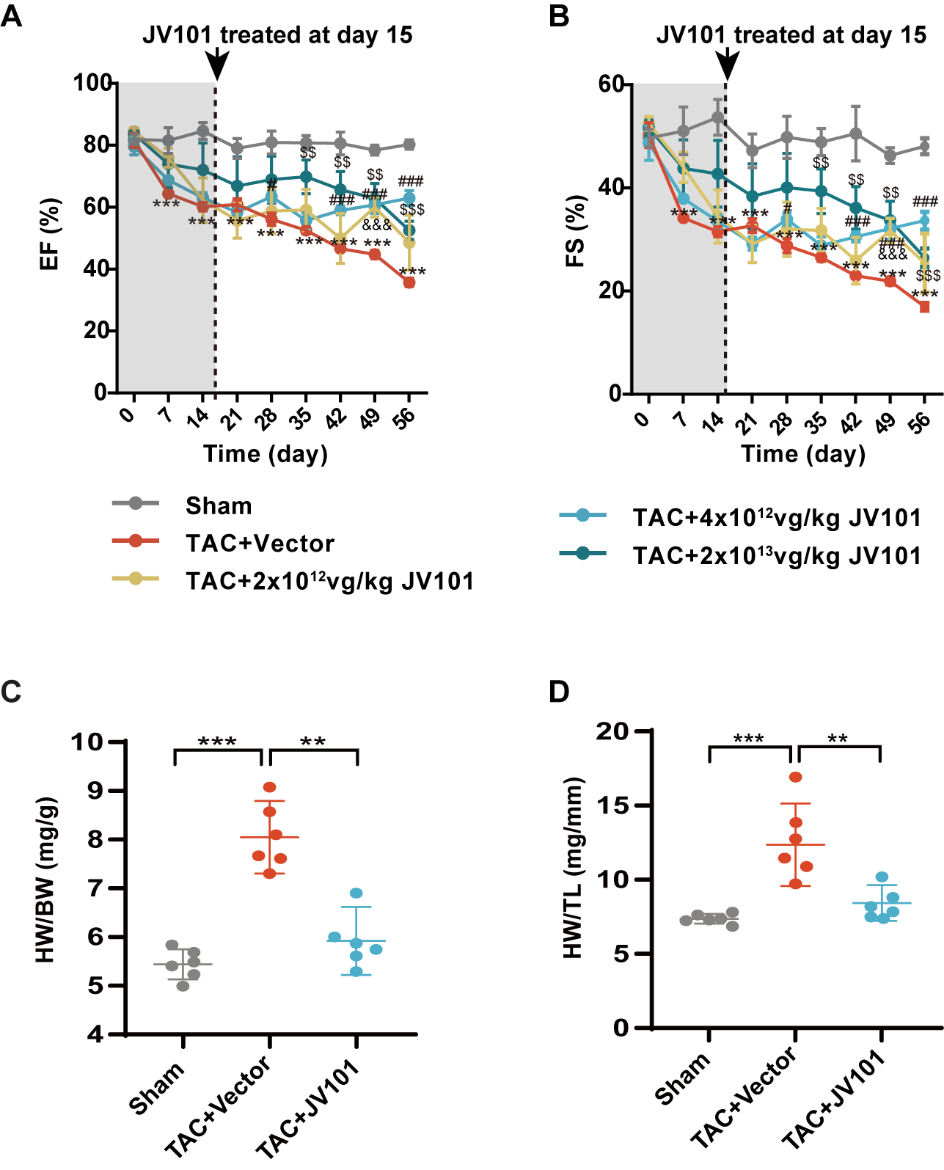


**Figure S2. JV101 dose titration and cardiac protection analysis in TAC mice.** (A and B) TAC animals that enter heart failure stage (decrease in LVEF by 20%) were randomly divided and intravenously injected on day 15 with escalating doses of JV101 (2×10^12^ vg/kg; 4 × 1012 vg/kg; 2×10^13^ vg/kg) or vector (2×10^13^ vg/kg). Cardiac function evaluated by echocardiography. Left ventricular ejection fraction (EF %, A) and fractional shortening (FS %, B) at 0, 7, 14, 21, 28, 35, 42, 49 and 56 days are shown. (Sham, n = 6; TAC + Vector, n = 8-10; TAC + JV101 2 × 10^12^ vg/kg, n = 5-6; TAC + JV101 4 × 10^12^ vg/kg, n = 8-10; TAC + JV101 2 × 10^13^ vg/kg, n = 5-6). Significance between TAC + Vector vs. saline [*]; 2 × 10^12^ vg/kg JV101 vs. TAC + Vector [&]; 4 × 10^12^ vg/kg JV101 vs. TAC + Vector [#]; 2 × 10^13^ vg/kg JV101 vs. TAC + Vector [$]. (C and D) Heart weight–to–body weight (HW/BW) and heart weight–to–tibia length (HW/TL) ratios at 56 days are shown (n = 6 animals each). Data are mean ± SEM. ^*^*P* < 0.05, ^**^*P* < 0.01, ^***^*P* < 0.001; ^&&&^*P* < 0.001; ^#^*P* < 0.05, ^###^*P* < 0.001; ^$$^*P* < 0.01, ^$$$^*P* < 0.01. Statistical differences among groups were analyzed by one-way ANOVA followed by Tukey’s multiple comparisons test.


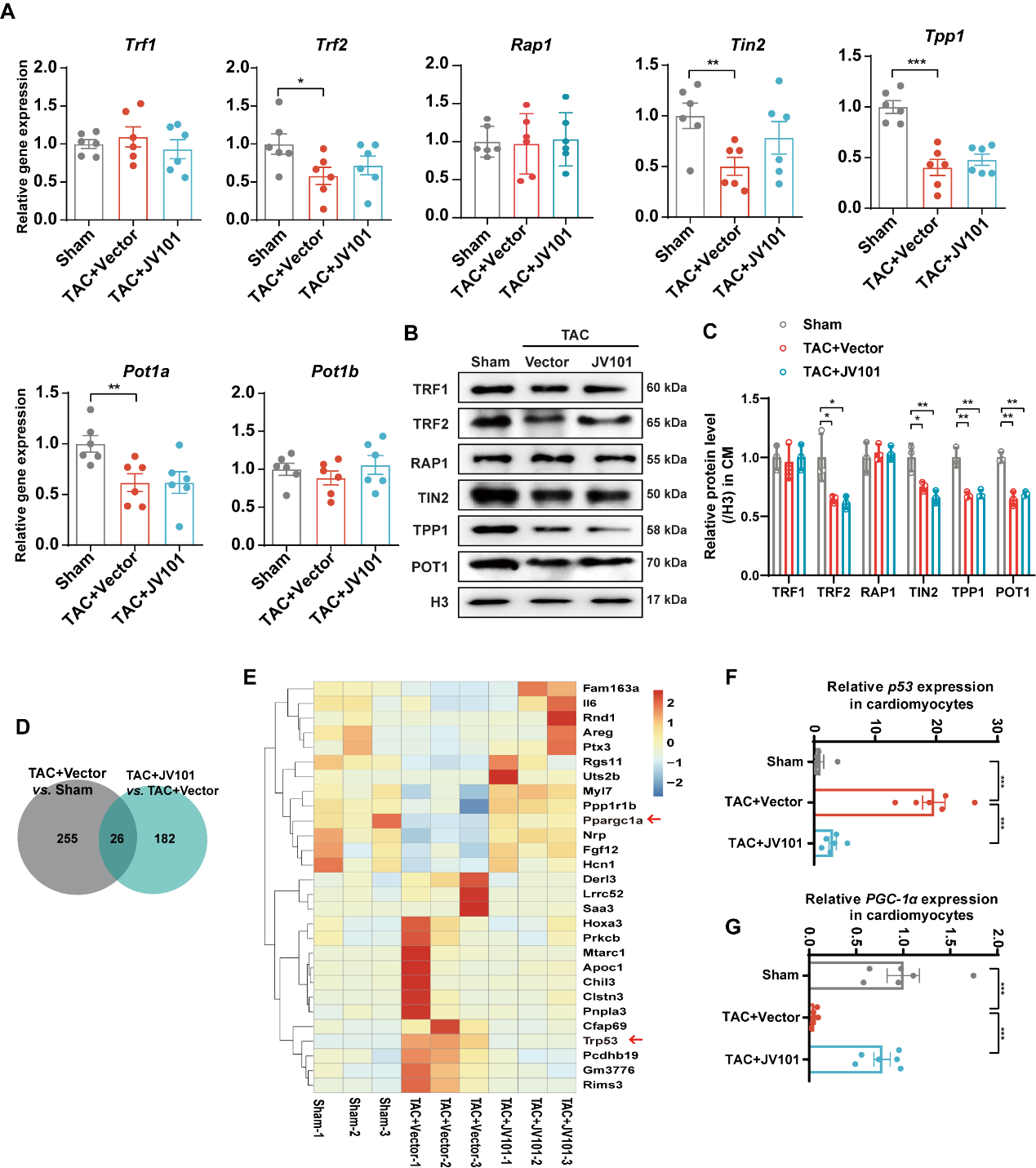


**Figure S3. JV101 failed to reverse the reduction of shelterin complex in cardiomyocytes perfused from TAC-induced heart failure mice and RNA-seq analysis.** (A) Primary cardiomyocytes were isolated from various groups of murine hearts. Endogenous expression levels of telomere capping genes were determined by RT-qPCR: *Trf1, Trf2, Tpp1, Tin2, Pot1* and *Rap1*. (n = 6 independent experiments). (B and C) Nuclear lysate isolated from cardiomyocytes perfused from mice of the TAC model was immunoblotted for TRF1, TRF2, RAP1, TIN2, TPP1 and POT1 and the quantitative analysis of the proteins. H3 was used as a loading control. (D) Venn diagram analysis of differentially expressed genes between TAC + Vector/Sham and TAC + JV101/TAC + Vector comparisons (n = 3). (E) Heat map analysis of 28 overlapping genes in different groups. (F and G) Relative p53 and PGC-1α mRNA level in cardiomyocytes perfused from mice of TAC + Vector, TAC + JV101 and Sham mice. Data are represented as mean ± SEM. ^*^*P* < 0.05, ^**^*P* < 0.01, ^***^*P* < 0.001. Statistical differences among groups were analyzed by one-way ANOVA followed by Tukey’s multiple comparisons test.


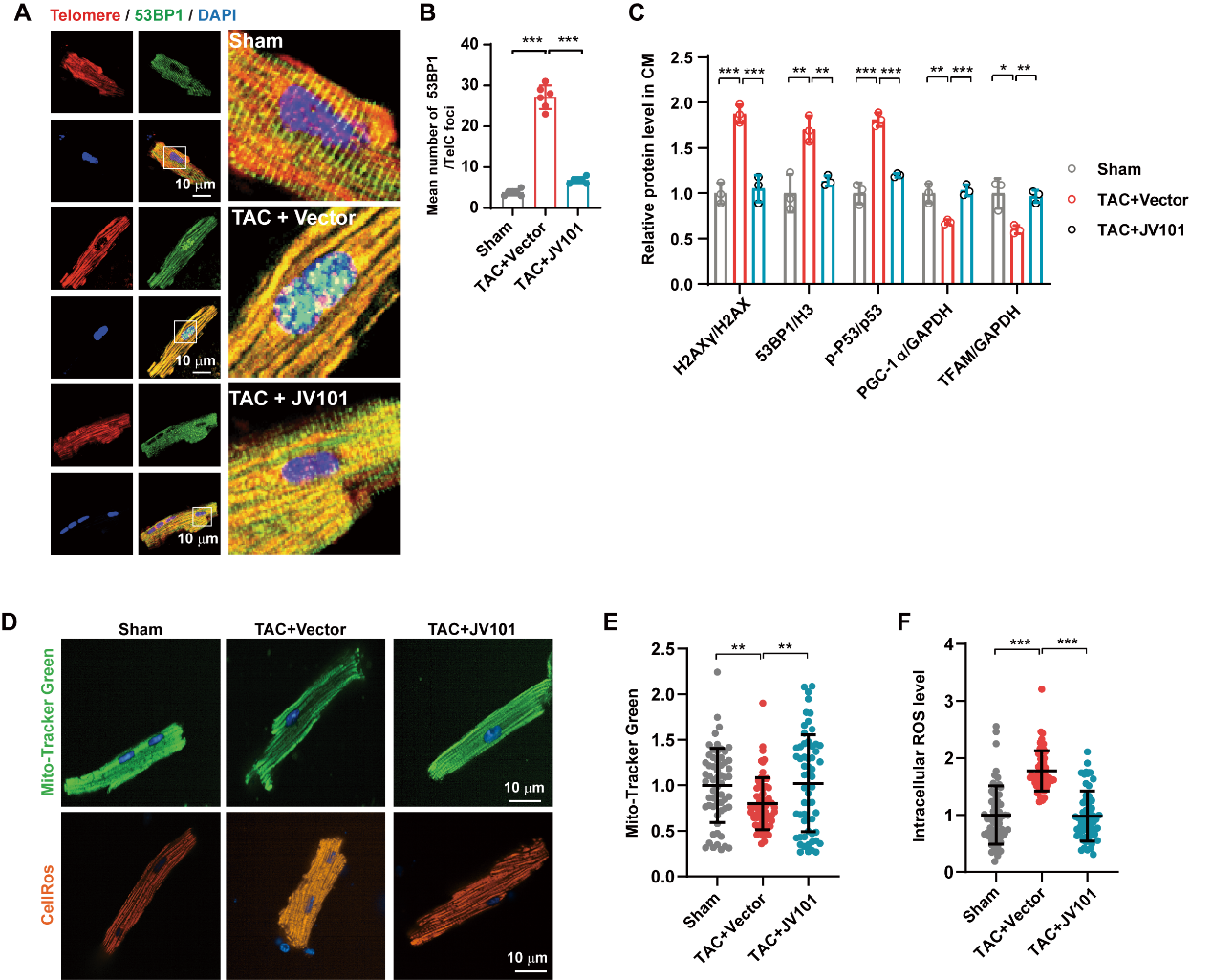


**Figure S4. JV101 decreased the DDR marker co-localization and reversed mitochondrial amount and cell ROS in cardiomyocytes perfused from TAC-induced heart failure mice.** (A and B) Representative micrograph showing accumulation of DNA damage response 53BP1 in cardiomyocytes perfused from TAC-induced heart failure group but not JV101 treatment group, followed by quantification. (C) Relative protein level of γH2AX, 53BP1, p-p53/p53, PGC-1α, TFAM in TAC + Vector, TAC + JV101 and Sham mice (Corresponds to Figure. 5D). (D-F) Representative confocal microscopy images of fluorescence staining for mitochondrial amount (Mito Tracker Green) and intracellular ROS (CellROS staining) in isolated cardiomyocytes from TAC cardiomyocytes. Quantification of mean mitochondrial amount (E) and cell ROS (F) fluorescence intensity (mice n = 3 per group; cardiomyocytes n = 60 per group). Data are represented as mean ± SEM. ^*^*P* < 0.05, ^**^*P* < 0.01, ^***^*P* < 0.001. Statistical differences among groups were analyzed by one-way ANOVA followed by Tukey’s multiple comparisons test.


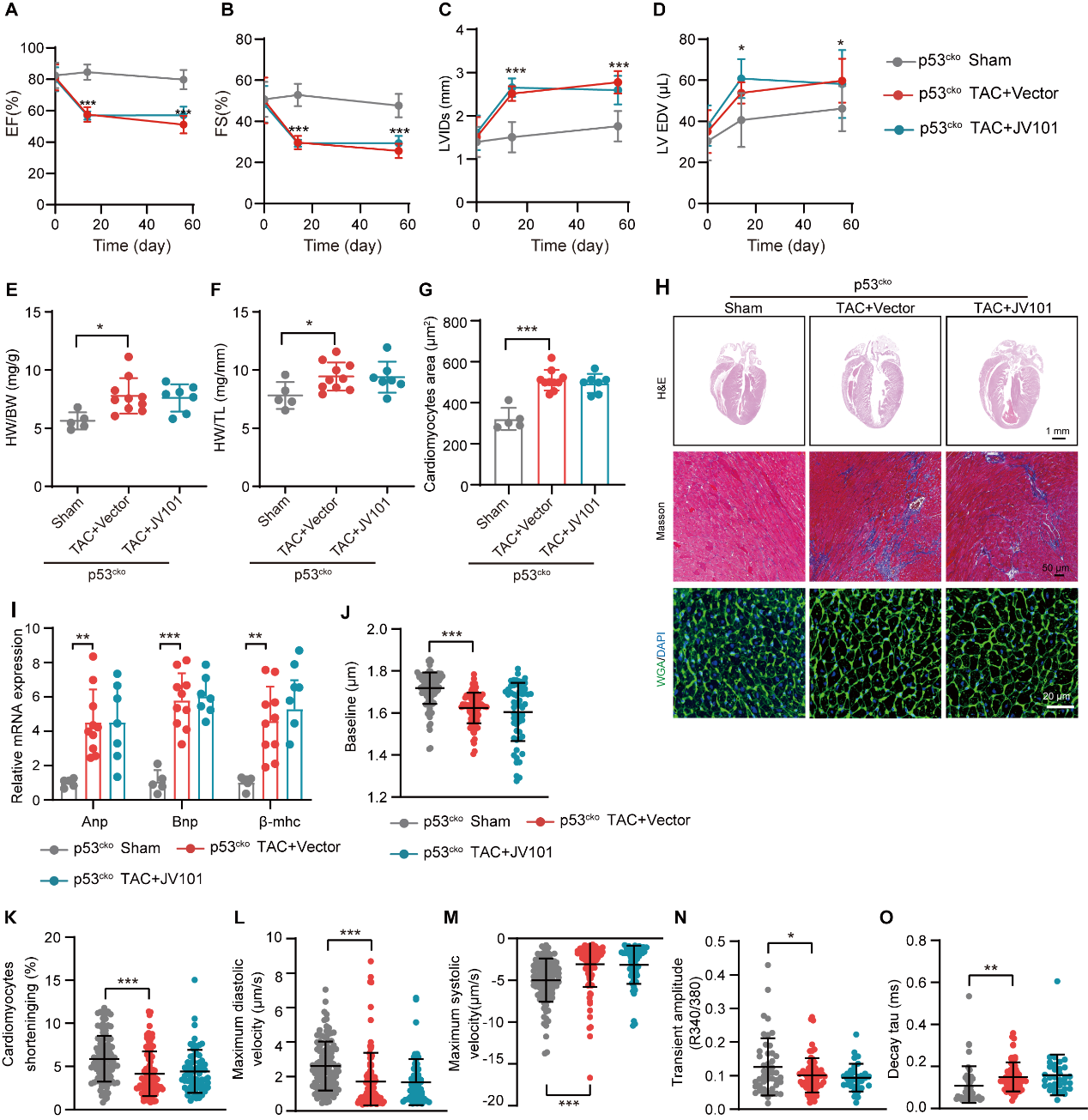


**Figure S5. JV101 didn’t reverse cardiac dysfunction in TAC-induced heart failure p53^cko^ mice.** (A-D) Cardiac function evaluated by echocardiography. Left ventricular ejection fraction (EF %), fractional shortening (FS %), left ventricular internal diameter at end-systole (LVIDs), and left ventricular end-diastolic volume (LV EDV) at 0, 14, and 56 days are shown. (p53^cko^ sham, n = 5; p53^cko^ TAC + Vector, n = 10; p53^cko^ TAC + JV101, n = 7; significance between p53^cko^ TAC + Vector vs. Sham [*] and p53^cko^ TAC + JV101 vs. p53^cko^ TAC + Vector [#] are shown). (E-F) Heart weight–to–body weight (HW/BW) (E) and heart weight–to–tibia length (HW/TL) (F) ratios at 56 days are shown. (H) Hematoxylin and eosin (longitudinal), Masson trichrome (longitudinal), and wheat germ agglutinin (transverse) micrographs are shown. (G) Quantitative analysis of cardiomyocyte cell sizes from transverse WGA staining (n =5-7 animals each). (I) Expression levels of *Anp, Bnp*, and *β-mhc* in primary cardiomyocytes were determined by RT-qPCR (cardiomyocytes from n = 5-10 animals each). (J-M) Quantification of sarcomere baseline, cardiomyocyte shortening, maximum diastolic velocity, and maximum systolic velocity of isolated cardiomyocytes are shown. (N and O) Quantification of calcium transient amplitude and decay tau are shown. Data are represented as mean ± SEM. ^*^*P* < 0.05, ^**^*P* < 0.01, ^***^*P* < 0.001. Statistical differences among groups were analyzed by one-way ANOVA followed by Tukey’s multiple comparisons test.


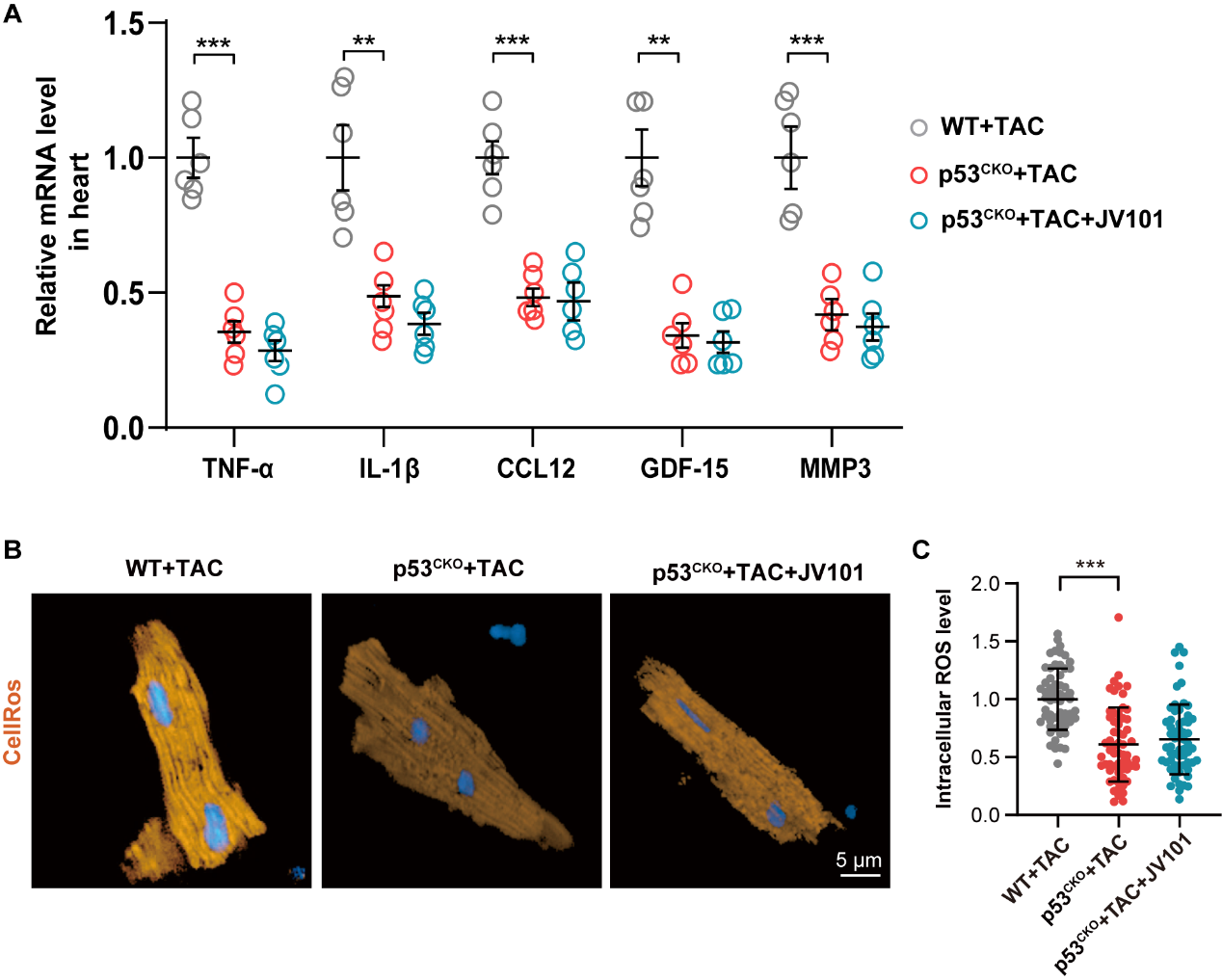


**Figure S6. Myocardial p53-deficiency (p53^CKO^) prevented TAC-induced inflammatory response and ROS.** (A) Expression levels of inflammatory cytokines in cardiomyocytes isolated from WT TAC group, p53^CKO^ TAC group, and JV101 treatment p53^CKO^ TAC group were determined by RT-qPCR (n = 6). (B and C) Representative confocal microscopy images of fluorescence staining for intracellular ROS (CellROS staining) in isolated cardiomyocytes. Quantification of mean ROS fluorescence intensities are shown (mice n = 3 per group; cardiomyocytes n = 60 per group). Data are represented as mean ± SEM. ***P* < 0.01, ****P* < 0.001. Statistical differences among groups were analyzed by one-way ANOVA followed by Tukey’s multiple comparisons test.


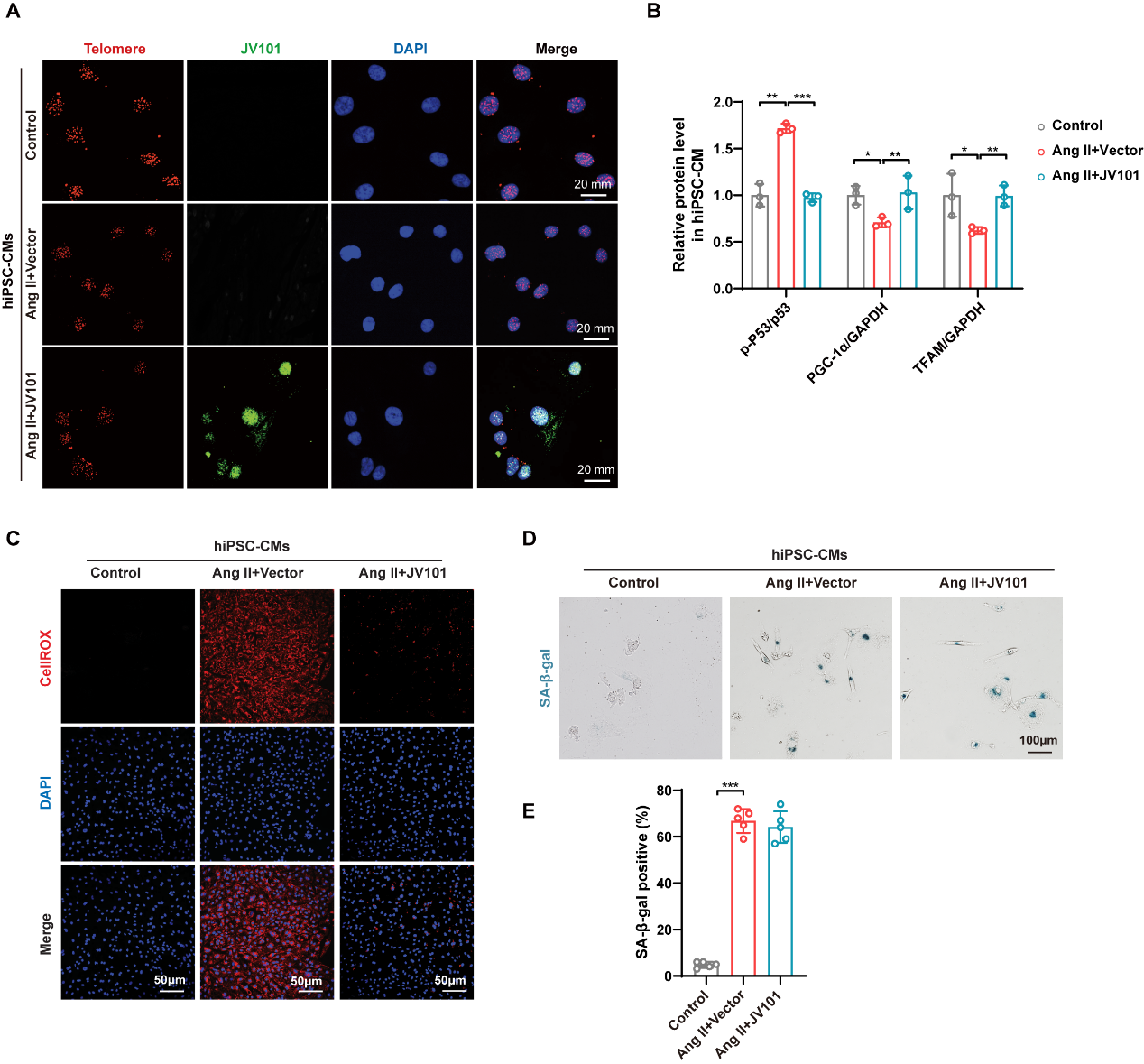


**Figure S7. JV101 reduced p53, restored PGC-1α and TFAM, and reduced cellular ROS in Ang II treated hiPSC-SMs.** (A) Representative micrographs of hiPSC-CMs displaying telomere Q-FISH (red) and nuclear modhTERT (green) after AAV9-modhTERT infection (DAPI [blue]). (B) Relative protein level of p-p53/p53, PGC-1α, and TFAM in hiPSC-SMs (Corresponds to Figure. 6D). (C) After treatment of 10 μM Ang II for 24 h, hiPSC-CMs were treated with JV101, representative micrographs of hiPSC-CMs showing CellROX at 72 h were shown. (D and E) Representative micrographs and quantification of SA-β-gal activity in control, Ang II+Vector, and Ang II+JV101 hiPSC-CMs. Data are represented as mean ± SEM. ^*^*P* < 0.05, ^**^*P* < 0.01, ^***^*P* < 0.001. Statistical differences among groups were analyzed by one-way ANOVA followed by Tukey’s multiple comparisons test.


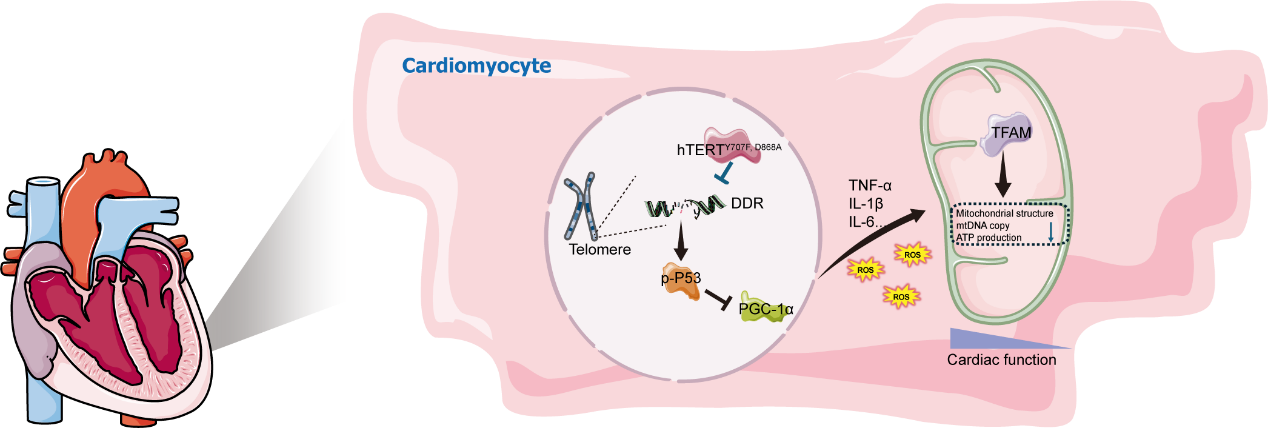


**Figure S8. Model of JV101 therapy in pressure overload–induced heart failure signaling.** The nuclear-localized and catalytic-null modhTERT^Y707F, D868A^ (JV101) inhibited telomeric DNA damage response (DDR), relieved p53-mediated PGC1α and TFAM suppression, blocked chronic inflammation and cell ROS, thereby rescuing mitochondrial structure abnormalities and dysfunction, and ultimately protected against heart failure.
